# New Aromatic Abietane Diterpenoids from *Lycopus europaeus* L. Fruits: ^1^H NMR Simulation-Aided Structure Elucidation and Enzyme Inhibition Screening

**DOI:** 10.3390/molecules31142441

**Published:** 2026-07-12

**Authors:** Marija S. Genčić, Danijela N. Nikolić, Jelena D. Živanović, Jelena M. Denić, Niko S. Radulović

**Affiliations:** Department of Chemistry, Faculty of Sciences and Mathematics, University of Niš, Višegradska 33, 18000 Niš, Serbia; danijela.nikolic1@pmf.edu.rs (D.N.N.); jelena.zivanovic@pmf.edu.rs (J.D.Ž.); aksicjelena9@gmail.com (J.M.D.)

**Keywords:** *Lycopus europaeus* fruits, aromatic abietanes, spectroscopic characterization, anticholinesterase activity, antiurease activity

## Abstract

The fruits of *Lycopus europaeus* L. represent an unusual source of highly oxygenated aromatic abietane diterpenoids. Following our previous identification of euroabienol (**1**) from this plant material, a phytochemical reinvestigation of the dichloromethane fruit extract was undertaken to characterize related minor constituents. Three new aromatic abietane diterpenoids, 4-epileonubiastrin (**2**), 3α-acetoxyeuroabienol (**3**), and 11-deoxyeuroabienol (**4**), were isolated together with euroabienol (**1**). Their structures and relative configurations were established by MS, HRMS, IR, and extensive 1D and 2D NMR analyses. Manual iterative full spin analysis of selected ^1^H NMR spin systems enabled refined determination of chemical shifts and coupling constants and provided additional support for conformational and configurational assignments, particularly in structurally congested parts of the molecules. To obtain a preliminary indication of biological relevance, compounds **1**–**4** and the semisynthetic *O*-methylated euroabienol derivative **5** were evaluated for acetylcholinesterase and urease inhibition. The observed effects were modest: compound **2** showed the highest AChE inhibition, reaching 31% at 50 μM, whereas compound **4** was the most active against jack bean urease, producing 40% inhibition at 100 μM. The study expands current knowledge of *L. europaeus* fruit diterpenoids and illustrates the value of ^1^H NMR simulation as a complementary tool in the elucidation of closely related abietane natural products.

## 1. Introduction

*Lycopus europaeus* L. (Lamiaceae), commonly known as gypsywort in English and *Vučja noga* or *gagalica* in Serbian, is a perennial species native to Europe and Asia and naturalized in parts of North America. The plant has attracted phytochemical and pharmacological interest because its extracts contain diverse phenolic constituents that have been associated with its medicinal properties [1,2]. In traditional use, *L. europaeus* has been described as an astringent, cosmetic, narcotic, and refrigerant plant [3]. Preparations obtained from the aerial parts have also been used in the management of mild hyperthyroidism, particularly when accompanied by vegetative nervous symptoms [4]. Experimental studies in rats further support effects of *L. europaeus* extracts on hyperthyroid-related manifestations, including cardiac signs and body temperature changes [5].

**Figure 1:**
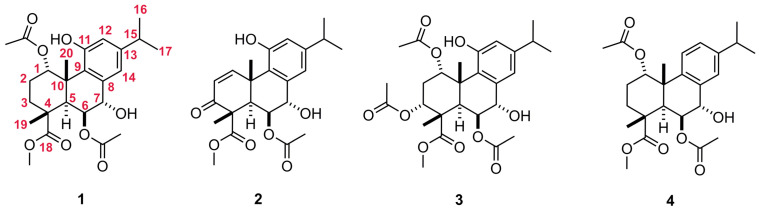
Structures of aromatic abietane diterpenoids **1**–**4** isolated from *L. europaeus* fruit extract. The carbon atom numbering scheme for the abietane skeleton is provided in structure **1**, where the carbon atom numbers are highlighted in red.

In our previous investigation of *L. europaeus* fruits, we identified euroabienol (**1**), an antimicrobial phenolic abietane diterpene with an unusual pattern of oxygenation in the aromatic C ring [6]. Its relatively high abundance in the fruit extract was unexpected because earlier studies of *L. europaeus* had mainly reported highly oxygenated isopimarane-type and aliphatic diterpenes from the aerial parts, especially leaves [7,8,9,10,11]. This organ-dependent difference in diterpene composition suggested that the fruits may represent an underexplored source of structurally distinctive abietane metabolites. Re-examination of the dichloromethane fruit extract by chromatographic fractionation, therefore, aimed to characterize the minor constituents related to euroabienol. This led to the isolation of three new aromatic abietane diterpenes, 4-epileonubiastrin (**2**), 3α-acetoxyeuroabienol (**3**), and 11-deoxyeuroabienol (**4**), together with the known compound **1** (Figure 1). Their structures were assigned by detailed MS, IR, and NMR spectroscopic analyses.

Aromatic abietanes are among the most widespread naturally occurring abietane diterpenoids and are commonly discussed in relation to chemical defense and bioactivity [12,13]. Several Lamiaceae-derived aromatic abietanes have also been reported to inhibit acetylcholinesterase (AChE), the enzyme responsible for hydrolysis of the neurotransmitter acetylcholine [14,15,16,17]. Since AChE inhibitors are clinically relevant in the symptomatic treatment of Alzheimer’s disease, where cholinergic dysfunction is a central feature, this enzyme remains a useful target for preliminary screening of natural products [18]. On this basis, compounds **1**–**4** were evaluated for their ability to inhibit AChE activity.

Urease inhibition was included as a second exploratory assay because a recent report identified abietatrien-3β-ol as an aromatic abietane with notable urease inhibitory activity [17]. Urease catalyzes the hydrolysis of urea into ammonia and carbamate and contributes to the pathogenicity of several ureolytic microorganisms, including *Helicobacter pylori*, *Proteus mirabilis*, and *Klebsiella pneumoniae* [19]. Although aromatic abietanes are well known for antimicrobial properties [12,13], urease inhibition within this structural class has been only rarely examined [17]. Urease activity has also been implicated in the pathogenesis of several antibiotic-resistant priority pathogens [20], maintaining interest in the discovery of new urease inhibitors [19,20]. Accordingly, compounds **1**–**4** were tested against jack bean urease as a widely used model enzyme for preliminary inhibitor screening, while recognizing that assays with bacterial ureases would be required to evaluate relevance to clinically important ureolytic pathogens.

## 2. Results and Discussion

### 2.1. Structure Elucidation of Aromatic Abietanes

**Figure 2:**
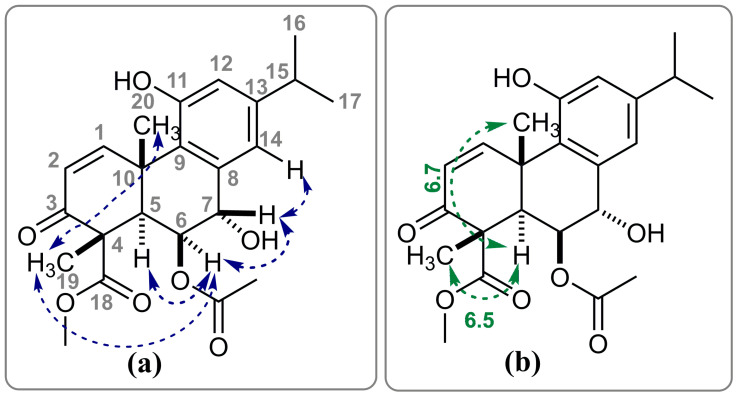
Key NOE interactions (**a**) and ^3^*J*_C–H_ coupling constants (in Hz; (**b**)) supporting the stereochemical assignment of compound **2**. Carbon atom numbers are shown in grey, NOE interactions are indicated by blue arrows, and C–H couplings and their corresponding ^3^*J*_C–H_ values are shown in green.

The dichloromethane extract of *L. europaeus* fruits was subjected to gradient dry-flash chromatography on SiO_2_, using mixtures from pure *n*-hexane to pure EtOAc. This separation first afforded two pure compounds from consecutive fractions eluted with 40% EtOAc, as indicated by TLC and GC–MS monitoring. The later-eluting compound was identified as euroabienol (**1**) by comparison of its MS and NMR data with those previously reported (Figures S1–S3) [6]. The earlier-eluting compound **2** showed spectral features closely related to those of leonubiastrin, an aromatic abietane differing from euroabienol by the presence of an α,β-unsaturated ketone in ring A and by configuration at C-4. The relative stereochemistry of leonubiastrin was originally established based on NOESY data, wherein axial H-5α exhibited NOE cross-peaks with H-6α and Me-18, while pseudoequatorial H-7β showed correlations with H-14 and Me-20 [21]. In contrast, analysis of the NOESY spectra of our abietane compound **2** revealed that Me-19 displayed NOE cross-peaks with axial Me-20 and pseudoequatorial H-6α, whereas axial H-5α showed a through-space interaction exclusively with H-6α (Figure 2a and Figure S8). Similar to euroabienol (**1**), H-7β in compound **2** exhibited NOE correlations with both H-6α and H-14.

Three-bond proton–carbon coupling constants (^3^*J*_C–H_) follow a Karplus-type relationship, analogous to proton–proton couplings, and are valuable for conformational and configurational assignments in saturated systems. In the non-decoupled ^13^C NMR spectrum of **2**, both Me-19 and Me-20 carbons appeared as quartets of doublets, with very similar ^1^*J*_C–H_ and ^3^*J*_C–H_ coupling constants (130.2 and 6.5 Hz for Me-19, and 130.0 and 6.7 Hz for Me-20, respectively; Figure 2b). The comparable ^3^*J*_C–H_ coupling constants of Me-19 and Me-20 carbons with axial H-5α imply that the corresponding dihedral angles (C19–C4–C5–H5α and C20–C10–C5–H5α) are essentially identical. A coupling constant of approximately 6.5 Hz is indicative of a dihedral angle close to 180°, consistent with a 1,3-diaxial relationship between Me-19, Me-20, and H-5α. Conversely, if Me-19 were α-oriented as in leonubiastrin, the dihedral angle would be approximately 60°, resulting in significantly smaller or undetectable three-bond C–H coupling constants [22].

Taken together, the NOESY correlations and the diagnostic long-range ^3^*J*_C–H_ coupling constants support assignment of compound **2** as the C-4 epimer of leonubiastrin. The compound is therefore designated 4-epileonubiastrin (**2**). The close similarity of the ^1^H and ^13^C NMR chemical shifts of **2** to those reported for leonubiastrin is not, by itself, configurationally diagnostic; the assignment rests primarily on the spatial information obtained from NOESY and on the long-range C–H coupling data from the non-decoupled ^13^C NMR spectrum. The configuration at C-4 is also consistent with that found in euroabienol (**1**), suggesting a plausible common biosynthetic relationship among these structurally related fruit metabolites.

**Figure 3:**
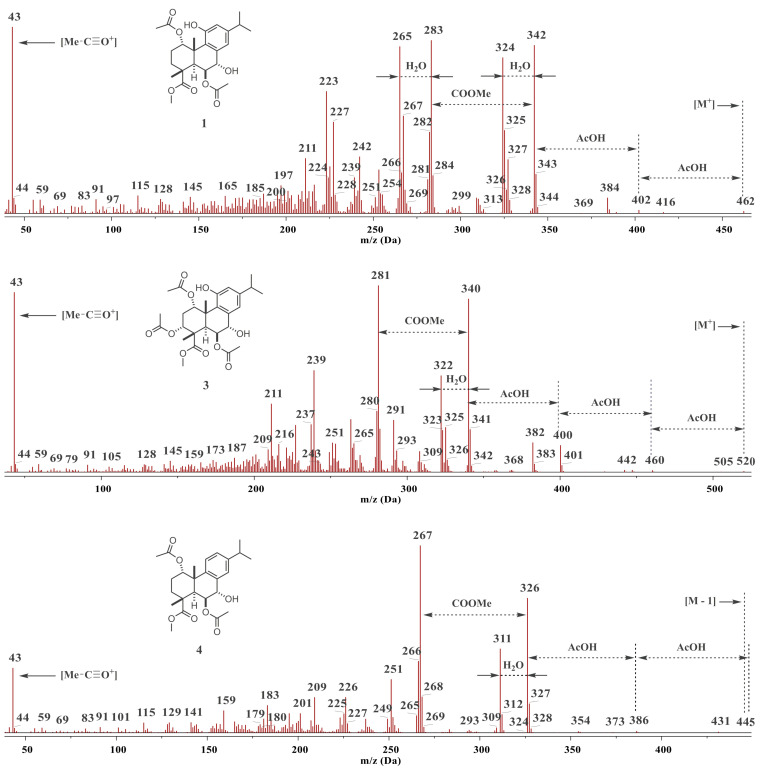
EI-MS spectra of abietanes **1**, **3**, and **4**, showing characteristic mass fragmentation patterns.

One polar fraction, eluted with 50% EtOAc (*v*/*v*), contained compound **3** as the major constituent, which exhibited the highest *m*/*z* value of 520 in its mass spectrum. The MS fragmentation pattern of compound **3** followed a characteristic sequence of consecutive acetic acid losses, resulting in the third most intense peak at [M – 3 × AcOH], indicating a triacetylated structure. This was further corroborated by the presence of *m*/*z* 43, which appeared as the second most intense peak (Figure 3 and Figure S9). This compound was obtained in pure form as a colorless amorphous solid after isocratic chromatography on SiO_2_. HRMS results suggested that the molecular formula of **3** is C_27_H_36_O_10_. The IR spectrum further corroborated the presence of ester (1750, 1727, 1707 cm^−1^), aromatic (1579 cm^−1^), and hydroxyl (3419 cm^−1^) functional groups (Figure S10).

Similar to euroabienol (**1**), the ^1^H and ^13^C NMR spectra of compound **3** (Table 1 and Table 2; Figures S11 and S12) revealed signals for an isopropyl group attached to an aromatic ring, two methyl groups attached to fully substituted *sp*^3^ carbon atoms, two aromatic protons in the *meta*-position, and a carbomethoxy group [6]. The most notable distinction was the presence of an additional acetoxyl group (δ_H_ 1.94, 3H, s; δ_C_ 169.7 and 21.1), as the NMR data indicated three acetoxyl groups in compound **3**, whereas compound **1** contained only two (Table 1 and Table 2). Furthermore, the addition of a few drops of D_2_O to the CDCl_3_ solution of compound **3** resulted in the disappearance of two signals in the ^1^H NMR spectrum: a sharp singlet at 6.21 ppm and a very broad signal at 2.18 ppm, suggesting the presence of two distinct hydroxyl groups. These two protons also gave rise to exchangeable cross-peaks in the NOESY spectrum with the same sign as the diagonal peaks, unlike the opposite sign seen in classical NOE cross-peaks.

**Figure 4:**
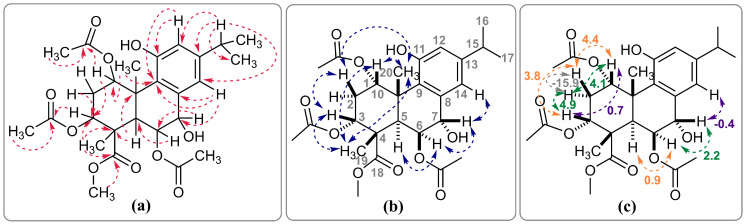
Key HMBC correlations (**a**), NOE interactions (**b**), and ^1^H–^1^H coupling constants (in Hz; (**c**)) supporting the structural assignment of compound **3**. Carbon atom numbers are shown in grey, HMBC correlations in red, NOE interactions in blue, and H–H couplings with their corresponding coupling constants in green, orange, violet, or light grey.

The coupling system of the (ROOC)CH–CH_2_–CH(COOR) (H-1, H-2α and 2β, and H-3, respectively) moiety [δ 6.13 (ddd, *J* = 4.4, 4.1, 0.7 Hz, 1H); 2.28 (ddd, *J* = −15.9, 4.9, 4.1 Hz, 1H); 2.37 (ddd, *J* = −15.9, 4.4, 3.8 Hz, 1H); 5.13 (dd, *J* = 4.9, 3.8, 0.7 Hz, 1H)] was determined through the ^1^H–^1^H COSY spectrum. The HMBC spectrum further confirmed correlations between the C-1 proton and the carboxyl carbon (δ 171.3) of one acetate group, as well as between H-3 and the carboxyl carbon (δ 169.7) of the other acetate group, establishing that the two acetates are positioned at C-1 and C-3 in the A ring, respectively (Figure 4a). The DEPT-135 experiment additionally confirmed the presence of a single methylene group (C-2) in the molecule (δ 27.2). ^1^H NMR simulation experiments were employed to accurately determine the chemical shifts and coupling constants of the protons associated with the described spin system. The low vicinal coupling constants (*J*(H_1_–H_2α or β_) and *J*(H_2α or β_–H_3_)), found to be all below 5 Hz, are consistent with gauche relationships. Accordingly, both H-1 and H-3 are likely (pseudo)equatorial and positioned above the plane, as these protons also exhibited NOE correlations with axial Me-19β and Me-20β, which themselves also display a mutual cross-peak (Figure 4b). Another spin system corresponding to the (C)_2_CH–CHOR–CHOH(C)_2_ fragment (H-5, H-6, H-7) of ring B, which is also present in compound **1**, was observed in the ^1^H–^1^H COSY spectrum as well. Chemical shifts and coupling constants were again precisely determined through simulation. The low values of vicinal coupling constants (below 2 Hz) indicate that these protons are nearly perpendicular to one another. This is consistent with the observation that axial H-5α exhibited NOE correlations exclusively with pseudoequatorial H-6α, while the pseudoequatorial H-7β proton showed NOE correlations with both H-6α and aromatic H-14. A long-range benzylic coupling between H-7β and H-14 was also observed in the ^1^H–^1^H COSY spectrum, with the ^4^*J* coupling constant of −0.4 Hz determined through simulation. These functionalities are consistent with the structure of the acetoxy derivative **3** of euroabienol (**1**), identified as 3α-acetoxyeuroabienol (or methyl 1α,3α,6β-triacetoxy-7α,11-dihydroxy-5α*H*-abieta-8,11,13-trien-18-oate), representing a novel diterpenoid. The key NOE and HMBC correlations, together with the diagnostic ^1^H–^1^H coupling constants, that were pivotal in the determination of the proposed structure are illustrated in Figure 4.

Another fraction, eluted with 30% EtOAc (*v*/*v*), drew attention due to the presence of one of its major constituents (**4**), whose MS fragmentation pattern, resembling that of abietane **1**, followed a characteristic sequence involving the successive loss of acetic acid and water molecules. This sequence resulted in the second and third most intense peaks in the spectrum, corresponding to [M − 2 × AcOH] and [M − 2 × AcOH − H_2_O], respectively (Figure 3 and Figure S13). This compound was isolated in pure form as a colorless amorphous solid following chromatography on Sephadex LH-20. High-resolution mass spectrometry (HRMS) analysis confirmed a molecular formula of this compound as C_25_H_34_O_7_. The molecular mass of compound **4** was 16 mass units lower than that of compound **1** and it represents a deoxygenated derivative of **1**. The IR spectrum was consistent with the presence of ester (1727 cm^−1^), aromatic (1577 cm^−1^), and hydroxyl (3420 cm^−1^) functional groups (Figure S14).

**Figure 5:**
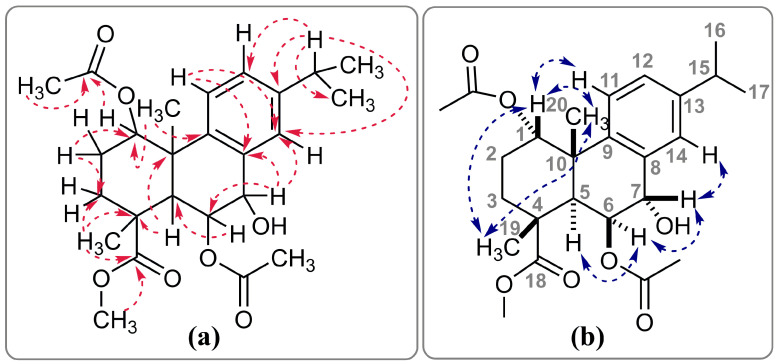
Important HMBC (**a**) and NOE (**b**) interactions of compound **4**. Carbon atom numbers are shown in grey, HMBC correlations in red, and NOE interactions in blue.

Its ^1^H NMR and ^13^C NMR spectra (Table 1 and Table 2; Figures S15 and S16) revealed the presence of an isopropyl group attached to a 1,2,4-trisubstituted benzene ring (δ_H_ 7.18 (d, *J_meta_* = 2.0 Hz, 1H), 7.14 (dd, *J_ortho_* = 8.3 Hz, *J_meta_* = 2.0 Hz, 1H), and 7.06 (d, *J_ortho_* = 8.3 Hz, 1H) and δ_C_ 129.0, 127.0 and 124.2, respectively), suggesting an aromatic abietatriene skeleton with no oxygen substituent on the aromatic C ring [23]. Additionally, similar to compound **1**, the spectra of **4** indicated the presence of a carbomethoxyl group, two acetoxyl groups and two methyl groups attached to fully substituted *sp*^3^ carbon atoms. The HMBC spectrum revealed a correlation between the C-1 proton and the carboxyl carbon (δ 170.8) of one of the acetates, as well as between the H-6 (δ 5.12) and the carboxyl carbon of the other acetate (δ 170.6), thereby confirming that the acetate groups are positioned at C-1 and C-6, respectively. Furthermore, the methoxyl protons (δ 3.76, s, 3H), along with H-5 (δ 3.18, d, *J* = 1.4 Hz, 1H) and the methyl protons at C-19 (δ 1.46, s, 3H), exhibited correlations with the carboxyl carbon at δ 178.0 (C-18), thereby confirming the partial structure of ring A and indicating that the COOMe group in compound **4** is attached to the C-4 position (Figure 5a). Compound **4** also exhibited other structural features closely resembling those of compound **1**, including the (C)_2_CH–CHOR–CHOH(C)_2_ (H-5, H-6, H-7) and (C)CH_2_–CH_2_–CH(C)(OR) (H-3α and 3β, H-2α and 2β, and H-1) closed spin systems [6], as evidenced by the ^1^H–^1^H COSY spectrum. The addition of a few drops of D_2_O to the CDCl_3_ solution of compound **4** resulted in the disappearance of only the broad signal at 2.66 ppm in the ^1^H NMR spectrum. These findings indicate that compound **4** constitutes an analog of compound **1**, with the only distinction being the absence of the phenolic group at position 11. Accurate determination of the chemical shifts and coupling constants for most protons in compound **4** was achieved through ^1^H NMR simulation experiments. However, the spin system of the (ROOC)CH–CH_2_–CH_2_–C fragment exhibited a splitting pattern too complex to be fully resolved using this approach.

The phenol group at the C-11 position in abietanes **1**–**3** induces a strong deshielding effect on the C-1 proton due to its coplanarity with the aromatic ring C and its proximity to the oxygen lone pairs of the C-11 hydroxyl group, resulting in a downfield shift in the C-1 proton resonance [6,21]. In contrast, the absence of the phenol group in abietane **4** causes the H-1 to exhibit a chemical shift (5.47 ppm) that is more akin to the H-1β in the analogous A ring of isopimarane diterpenes previously isolated from this species (4.84–4.97 ppm) [10,11]. The stereochemistry presented in structure **4** was determined based on the NOESY spectrum. The axial H-5α proton exhibited NOE cross-peaks with the H-6α and H-3α protons (δ 2.17), while the pseudoequatorial H-7β proton showed NOE interactions with the H-14 and pseudoequatorial H-6α protons. NOESY correlations between Me-19 and Me-20, along with their respective interactions with H-1β, were key to determining the relative configurations of the chiral centers in ring A (Figure 5b). The relative stereochemistry of compound **4** is consistent with that of compound **1**, thereby confirming the identity of the compound as 11-deoxyeuroabienol, or methyl 1α,6β-diacetoxy-7α-hydroxy-5α*H*-abieta-8,11,13-trien-18-oate, representing a newly characterized diterpenoid.

As in our previous study, the multiplicity of several signals in the ^1^H NMR spectrum remained unresolved [6]; therefore, manual iterative full spin analysis was employed to accurately determine the chemical shifts and coupling constants of euroabienol (**1**). With the exception of a strong axial–axial vicinal coupling between H-2β and H-3α (^3^*J* = 13.7 Hz), all other vicinal coupling constants were below 5 Hz. In addition to a benzylic long-range coupling between H-7β and H-14 (^4^*J* = −0.6 Hz), a W-type long-range interaction was observed between the (pseudo)equatorial protons H-1β and H-3β (^4^*J* = 0.6 Hz). Moreover, re-examination of the ^13^C NMR spectrum revealed a typographical error in the previously reported chemical shift in the methyl carbon C-18 of the COOMe group. The correct value is 52.5 ppm, rather than 42.4 ppm as reported earlier [6].

Compound **5** (methyl 1α,6β-diacetoxy-7α-hydroxy-11-methoxy-5α*H*-abieta-8,11,13-trien-18-oate) was obtained by O-methylation of compound **1** using MeI in the presence of K_2_CO_3_ to evaluate the influence of the phenolic hydrogen-bond donor on bioactivity. This semisynthetic derivative was characterized by MS, IR, and NMR spectroscopy (Figures S17–S20). A +14 amu shift in the molecular ion (*m*/*z* 476) relative to **1**, disappearance of the phenolic proton signal (~6 ppm) and appearance of a methoxy singlet at 3.75 ppm in the ^1^H NMR spectrum, together with a new ^13^C resonance at 55.0 ppm, confirmed formation of the O-methylated euroabienol derivative.

### 2.2. Biological Activities of Aromatic Abietanes

**Figure 6:**
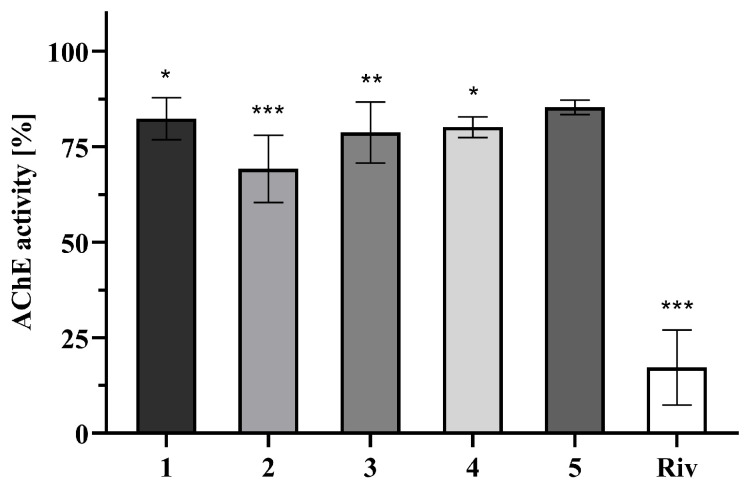
Anti-AChE effect of abietanes **1**–**5** and rivastigmine (Riv) at a concentration of 50 µM. Data are expressed as mean ± SD of three independent experiments (*n* = 3), each performed in technical triplicate. Statistical analysis was performed using one-way ANOVA followed by Dunnett’s multiple comparisons test versus the negative control (MeOH, final concentration 1%). *** *p* < 0.001, ** *p* < 0.01, and * *p* < 0.05 indicate statistically significant differences compared to the control.

The AChE inhibitory activities of compounds **1**–**4** and the *O*-methylated derivative **5** were evaluated using a modified Ellman assay [24]. All compounds were tested at 50, 20, and 4 μM. At 50 μM, compounds **1**–**4** produced statistically significant but modest inhibition of AChE activity, within the range of approximately 20–31% (Figure 6), whereas no detectable inhibition was observed at 20 or 4 μM. Higher test concentrations were not experimentally accessible because of limited solubility in the assay medium. Consequently, inhibition did not reach 50% and IC_50_ values could not be determined for the test compounds. Under the same conditions, the reference inhibitor rivastigmine reduced AChE activity by 83% at 50 μM, and its experimentally determined IC_50_ value of 10.2 μM was consistent with literature values [25,26].

The limited activity range does not allow reliable structure–activity conclusions for AChE inhibition. Earlier studies suggested that an aromatic C ring and the absence of oxygenation at C-3 may contribute to AChE inhibition in related abietanes [15], whereas abietatrien-3β-ol has been reported as more active than galantamine (IC_50_ = 4.4 μM) [17]. In the present series, euroabienol (**1**) and its 3α-acetoxy derivative **3** showed similar effects, while O-methylation of the phenolic group did not improve activity. Compound **2** was the most active member of the series and contains an α,β-enone moiety in ring A; however, the small number of compounds and modest inhibition levels preclude assigning a specific role to this structural feature.

**Figure 7:**
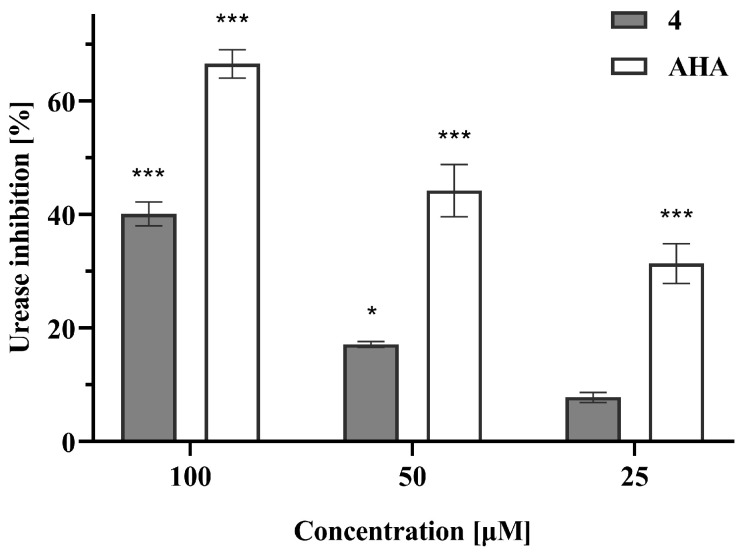
Inhibition of urease activity by 11-deoxyeuroabienol (**4**) compared to the reference inhibitor acetohydroxamic acid (AHA). Data are expressed as mean ± SD of three independent experiments (*n* = 3), each performed in technical triplicate. Statistical analysis was performed using one-way ANOVA followed by Dunnett’s multiple comparisons test versus the negative control (EtOH, final concentration 4.6%). *** *p* < 0.001 and * *p* < 0.05 indicate statistically significant differences compared to the control.

The effects of compounds **1**–**5** on urease activity were assessed using the indophenol/Berthelot colorimetric method with jack bean urease and urea as substrate [27,28,29]. This assay follows formation of the blue indophenol complex derived from ammonium ions released during enzymatic urea hydrolysis. Each compound was tested at 25, 50, and 100 μM. Among the tested compounds, only 11-deoxyeuroabienol (**4**) displayed a clear dose-dependent inhibitory effect, reaching 40% inhibition at 100 μM (Figure 7). Further concentration increases were precluded by solubility limitations, and an IC_50_ value could therefore not be determined. Under identical assay conditions, the reference inhibitor acetohydroxamic acid (AHA) produced 67% inhibition at 100 μM, with an IC_50_ value of 61.1 μM, in agreement with literature data [29].

Compound **4** differs from the other tested abietanes by lacking a hydroxyl or methoxy substituent on the aromatic C ring. A similar absence of aromatic oxygenation is present in abietatrien-3β-ol, which has been reported as a potent urease inhibitor (IC_50_ = 16.1 μM or 4.61 μg/mL) [17]. Nevertheless, the present dataset is too limited to determine whether this feature directly governs urease inhibition. The results should therefore be interpreted as preliminary screening data showing that subtle changes in oxygenation within related aromatic abietanes may be reflected in different enzyme responses, rather than as a defined structure–activity relationship.

Overall, these results indicate weak-to-moderate, target-dependent inhibitory effects of selected aromatic abietane diterpenes against AChE and jack bean urease under the tested conditions. Differences in inhibition were observed among the tested metabolites, although the limited dataset does not allow definitive conclusions regarding structural determinants of activity.

## 3. Materials and Methods

### 3.1. General Experimental Procedures

All commercially available solvents and chemicals were used as received without further purification. Chromatographic separations were carried out using silica gel 60 (particle size 40–63 µm; Merck, Darmstadt, Germany). The progress of chromatographic separations was monitored by thin-layer chromatography (TLC) on aluminum plates precoated with silica gel 60 F_254_ (Merck, Darmstadt, Germany). Visualization was performed under UV light (254 nm) and/or by spraying the plates with a 1:1 (*v*/*v*) aqueous sulfuric acid solution, followed by brief heating.

^1^H NMR (400 MHz, including selective homonuclear decoupling experiments), ^13^C NMR (100.6 MHz), DEPT-90, DEPT-135, NOESY, and gradient ^1^H–^1^H COSY, HSQC, and HMBC spectra were recorded on a Bruker Avance III 400 MHz spectrometer (Bruker, Fällanden, Switzerland), equipped with a 5 mm dual ^13^C/^1^H probe. All NMR spectra were acquired at 20 °C in deuterated chloroform (CDCl_3_), with chemical shifts (δ) reported in parts per million (ppm), referenced to TMS. Scalar couplings (*J*) are reported in Hertz (Hz). These 1D and 2D NMR experiments were performed using standard Bruker built-in pulse sequences.

Gas Chromatography-Mass Spectrometry (GC-MS) was employed to monitor chromatographic separations and assist in the preliminary identification of fraction compositions. GC-MS analyses were conducted using a Hewlett-Packard 6890N gas chromatograph (Agilent Technologies, Santa Clara, CA, USA), equipped with a fused silica capillary column (SLB-5MS, 5% diphenyl/95% dimethylpolysiloxane, 30 m × 0.25 mm, 0.25 μm film thickness; Merck, Darmstadt, Germany) and coupled to a 5975B mass selective detector (Agilent Technologies, Santa Clara, CA, USA). The injector and interface were operated at 250 and 300 °C, respectively. The oven temperature was raised from 70 to 315 °C at a heating rate of 5 °C/min, and the program ended with an isothermal period of 30 min. Helium was used as a carrier gas at 1.0 mL/min. The samples, 1 μL of the sample solutions in Et_2_O (1 mg dissolved in 1 mL), were injected in a splitless mode. The mass selective detector was operated at the ionization energy of 70 eV, in the 35−750 amu range, and at a scanning speed of 0.34 s. High-resolution mass spectrometry (HRMS) was performed using a JEOL JMS-700 mass spectrometer (Jeol, Peabody, MA, USA) with an ionization energy of 70 eV, an ionization trap current of 300 μA, and a source temperature of 230 °C. Infrared (IR) spectra were recorded using an ATR (attenuated total reflectance) technique on a Thermo Nicolet 6700 FTIR spectrometer (Thermo Fisher Scientific, Waltham, MA, USA).

### 3.2. Plant Material

Fruits of *L. europaeus* were collected on 28 October 2024 from wetland areas in the western part of Niš, Serbia. Voucher specimens were deposited in the Herbarium of the Faculty of Science and Mathematics, University of Niš (No. MG1224).

### 3.3. Extraction and Isolation

Freshly collected fruits of *L. europaeus* (30 g, wet weight) were finely ground and extracted with dichloromethane (300 mL) using an ultrasonic bath (Elma Transsonic TI-H-5, MF3, 35/130 kHz, 230 V (Elma, Singen, Germany)) operated at 35 kHz for 2 h at room temperature (rt). The resulting crude extract (approximately 4 g) was concentrated by solvent evaporation and then purified using dry-flash column chromatography on silica gel (40–63 µm), with *n*-hexane-EtOAc mixtures of increasing polarity as the mobile phase, resulting in 18 fractions. Fractions were pooled based on TLC and GC-MS analysis. Fraction 13, eluted with *n*-hexane-EtOAc (3:2, *v*/*v*), yielded pure compound **2** (21.3 mg). Fractions 14 and 15, eluted with the same mobile phase, afforded crystalline compound **1** (151.1 mg). Fraction 10 (115 mg), eluted with *n*-hexane-EtOAc (7:3, *v*/*v*), contained compound **4** as one of the main constituents. This fraction was further purified by rechromatography on a Sephadex LH20 column (Cytiva, Marlborough, MA, USA), using MeOH-CHCl_3_ (1:1, *v*/*v*) as the eluent, resulting in pure compound **4** (22.2 mg). Fractions 16 and 17 (189 mg), eluted with *n*-hexane-EtOAc (1:1, *v*/*v*), predominantly contained compound **3**. These two fractions were rechromatographed using isocratic column chromatography on SiO_2_ with *n*-hexane-Et_2_O (1:3, *v*/*v*), yielding pure compound **3** (30.4 mg).

Euroabienol (**1**) [methyl 1α,6β-diacetoxy-7α,11-dihydroxy-5α*H*-abieta-8,11,13-trien-18-oate]: retention time (R_t_) = 42.497 min (SLB-5MS column); MS (EI), *m*/*z* (%) 462 (1.3) [M^+^], 402 (1.8) [M − AcOH], 384 (8.4) [M − AcOH − H_2_O], 342 (90.2) [M − 2 × AcOH], 324 (83.6) [M − 2 × AcOH − H_2_O], 309 (8.4) [M − 2 × AcOH − H_2_O − CH_3_], 283 (92.9) [M − 2 × AcOH − COOCH_3_], 265 (89.6) [M − 2 × AcOH − COOCH_3_ − H_2_O], 253 (23.5), 242 (30.5), 223 (65.5), 211 (29.2), 197 (15), 165 (9.4), 145 (8.9), 128 (7.7), 115 (9.6), 91 (7.6), 77 (4.1), 69 (3.9), 43 (100).

4-Epileonubiastrin (**2**) [methyl 6β-acetoxy-7α,11-dihydroxy-3-oxo-5α*H*-abieta-1,8,11,13-tetraen-18-oate]: retention time (R_t_) = 40.941 min (SLB-5MS column); FTIR (neat, cm^−1^) 3416 (w, *ν*(O–H)), 2929 (w, *ν*(CH_2_)_as_), 1727 (s, *ν*(C=O)_ester_), 1679 (s, *ν*(C=O)_ketone_), 1434 (m), 1368 (m), 1237 (s, *ν*(C–O)), 1029 (m), 913 (m), 755 (s), 723 (s); MS (EI), *m*/*z* (%) 416 (16.3) [M^+^], 356 (4.7) [M − AcOH], 341 (12.1), 324 (3.5), 309 (11.9), 297 (48.3) [M − AcOH − COOCH_3_], 281 (20.8), 267 (5.1), 253 (7.3), 241 (11.8), 227 (87), 215 (41), 202 (62), 187 (32.5), 173 (13.3), 147 (9.9), 129 (13.5), 115 (13.1), 105 (13.8), 91 (11.3), 77 (6), 69 (7.7), 43 (100); HRMS (EI) calcd for C_23_H_28_O_7_^+^: 416.1830, found 416.1836.

3α-Acetoxyeuroabienol (**3**) [methyl 1α,3α,6β-triacetoxy-7α,11-dihydroxy-5α*H*-abieta-8,11,13-trien-18-oate]: retention time (R_t_) = 45.774 min (SLB-5MS column); FTIR (neat, cm^−1^) 3419 (w, *ν*(O–H)), 2958 (w, *ν*(CH_3_)_as_), 1750, 1727, and 1707 (s, *ν*(C=O)_ester_), 1579 (w, *ν*(C=C)), 1362 (m), 1227 (s, *ν*(C–O)), 1054 (m), 986 (m), 863 (m), 656 (m); MS (EI), *m*/*z* (%) 520 (0.5) [M^+^], 505 (0.2) [M − CH_3_], 502 (0.2) [M − H_2_O], 460 (1) [M − AcOH], 442 (1.1) [M − AcOH – H_2_O], 400 (14.4) [M − 2 × AcOH], 382 (15.9) [M − 2 × AcOH − H_2_O], 340 (92.8) [M − 3 × AcOH], 325 (24.1) [M − 3 × AcOH − CH_3_], 322 (51.8) [M − 3 × AcOH − H_2_O], 291 (28), 281 (100) [M − 3 × AcOH − COOCH_3_], 263 (28.4) [M − 3 × AcOH − COOCH_3_ − H_2_O], 251 (15.8), 239 (54.6), 227 (25.2), 211 (36.7), 199 (8.4), 165 (5.3), 145 (6), 128 (4.2), 115 (3.7), 105 (2.9), 91 (3.9), 77 (2), 59 (4.3), 43 (96.3); HRMS (EI) calcd for C_27_H_36_O_10_^+^: 520.2303, found 520.2308.

11-Deoxyeuroabienol (**4**) [methyl 1α,6β-diacetoxy-7α-hydroxy-5α*H*-abieta-8,11,13-trien-18-oate]: retention time (R_t_) = 39.898 min (SLB-5MS column); FTIR (neat, cm^−1^) 3421 (w, *ν*(O–H)), 2957 (w, *ν*(CH_3_)_as_), 2932 (w, *ν*(CH_2_)_as_), 1727 (s, *ν*(C=O)_ester_), 1577 (w, *ν*(C=C)), 1371 (m), 1236 (s, *ν*(C–O)), 1026 (s), 946 (m), 828 (m); MS (EI), *m*/*z* (%) 445 (0.1) [M − H], 431 (0.5) [M − CH_3_], 386 (0.9) [M − AcOH], 368 (0.1) [M − AcOH − H_2_O], 354 (0.8), 326 (71.8) [M − 2 × AcOH], 311 (44.6) [M − 2 × AcOH − CH_3_], 283 (1.8), 267 (100) [M − 2 × AcOH − COOCH_3_], 251 (28.3), 249 (7.1) [M − 2 × AcOH − COOCH_3_ − H_2_O], 226 (18.7), 209 (18.6), 195 (10), 183 (14.3), 159 (11.5), 141 (5.3), 129 (5.2), 115 (5.1), 105 (1.9), 91 (2.8), 77 (1.1), 59 (2.5), 43 (34.4); HRMS (EI) calcd for C_25_H_34_O_7_^+^: 446.2300, found 446.2307.

The NMR data of compounds **1**–**4** are presented in Table 1 and Table 2. MS, IR and NMR spectra of compounds **1**–**4** are given in Supplementary Materials (Figures S1–S16).

### 3.4. Synthesis of O-Methylated Euroabienol (***5***)

To stirred suspension of anhydrous K_2_CO_3_ (69 mg, 0.50 mmol) in dry acetone (5 mL), a solution of euroabienol (**1**) (46.2 mg, 0.10 mmol) in dry acetone (2 mL) was added dropwise at rt. After 35 min, MeI (31 μL, 0.50 mmol) was added dropwise and the mixture was heated at reflux for 2 h, then stirred overnight at rt. The reaction mixture was poured into water and extracted with Et_2_O. The combined organic phases were washed with water, dried (MgSO_4_), filtered, and concentrated to afford compound **5** (42.8 mg, 90% yield). The product was obtained sufficiently pure and was used without further purification. Its purity was verified by ^1^H/^13^C NMR and GC-MS analyses. The structure of compound **5** was confirmed by ^1^H/^13^C NMR, IR and MS; corresponding spectra are provided in the Supplementary Materials (Figures S17–S20).

*O*-Methylated euroabienol (**5**) [methyl 1α,6β-diacetoxy-7α-hydroxy-11-methoxy-5α*H*-abieta-8,11,13-trien-18-oate]: retention time (R_t_) = 41.210 min (SLB-5MS column); FTIR (neat, cm^−1^) 3458 (w, *ν*(O–H)), 2958 (w, *ν*(CH_3_)_as_), 1727 (s, *ν*(C=O)_ester_), 1612 (w), 1573 (w, *ν*(C=C)), 1369 (m), 1235 (s, *ν*(C–O)), 1026 (s), 946 (m), 828 (m); MS (EI), *m*/*z* (%) 476 (4.8) [M^+^], 458 (0.1) [M − H_2_O], 416 (1.6) [M − AcOH], 384 (0.7), 356 (65.6) [M − 2 × AcOH], 341 (64) [M − 2 × AcOH − CH_3_], 324 (1.6), 313 (2.7), 297 (100) [M − 2 × AcOH − COOCH_3_], 281 (33.7), 256 (25.9), 239 (22.9), 225 (13.6), 211 (9.1), 189 (17.6), 173 (4.9), 144 (7.2), 128 (4.2), 115 (8.4), 91 (5.1), 77 (1.5), 69 (1.5), 55 (4.6), 43 (66.8); ^1^H NMR (CDCl_3_) δ 6.82 (br d, *J* = 1.80 Hz, 1H, H-14), 6.63 (d, *J* = 1.80 Hz, 1H, H-12), 6.16 (m, 1H, H-1), 5.03 (m, 1H, H-6α), 4.47 (br d, *J* = 2.20 Hz, 1H, H-7β), 3.75 (br s, 6H, overlapped 18-COOMe and 11-OMe), 3.19 (m, 1H, H-5α), 2.85 (septet, *J* = 6.9 Hz, 1H, H-15), 2.33 (br s, 1H, 7α-OH), 2.14* (m, 1H, H-3α), 2.11* (m, 1H, H-2β), 2.02 (s, 3H, 6β-OC(O)Me), 1.93 (m, 1H, H-2α), 1.74 (s, 3H, 1α-OC(O)Me), 1.68 (s, 3H, Me-20β), 1.48* (m, 1H, H-3β), 1.45 (s, 3H, Me-19β), 1.24 (br d, *J* = 6.9 Hz, 6H, Me-16 and Me-17); ^13^C NMR (CDCl_3_): δ 178.1 (C-18), 170.5 (1α-OC(O)Me or 6α-OC(O)Me), 170.4 (1α-OC(O)Me or 6β-OC(O)Me), 156.9 (C-11), 148.3 (C-13), 135.9 (C-8), 129.1 (C-9), 121.5 (C-14), 109.1 (C-12), 75.2 (C-6), 74.2 (C-1), 70.4 (C-7), 55.0 (11-OMe), 52.4 (18-COOMe), 47.7 (C-4), 42.7 (C-10), 37.5 (C-5), 33.7 (C-15), 31.3 (C-3), 23.9 (Me-16 or Me-17), 23.6 (Me-16 or Me-17), 22.2 (C-2), 21.8 (Me-20β), 21.5 (1α-OC(O)Me or 6β-OC(O)Me), 21.1 (1α-OC(O)Me or 6β-OC(O)Me), 18.3 (Me-19β); Chemical shifts marked with an asterisk (*) were estimated from observed HSQC correlations due to signal overlap; HRMS (EI) calcd for C_26_H_36_O_8_^+^: 476.2405, found 476.2409.

### 3.5. ^1^H NMR Full Spin Analysis

^1^H NMR full spin analysis was performed by manually adjusting δ_H_ and *J* values according to the general principles of iterative full spin analysis described previously [30,31], to fit the experimentally available values and further optimized using the Spin Simulation module implemented in MestReNova version 14.1.2 (Mestrelab Research, Santiago de Compostela, Spain). This iterative procedure led to a systematic refinement of all calculated NMR parameters until the simulation outcome was in excellent agreement (normalized root mean square deviation (NRMSD) < 0.05%) with the experimental data of the isolated compounds. The reported δ_H_ and *J* values represent optimized parameters derived from the final spectral fit; therefore, the number of decimal places reflects the precision of the simulation rather than the absolute experimental accuracy of the measurements.

### 3.6. Biological Activity Assessment

#### 3.6.1. AChE (Acetylcholinesterase) Inhibitory Activity

The AChE inhibitory activities of compounds **1**–**5** were assessed using a quantitative colorimetric assay based on Ellman’s method [24]. In brief, 25 µL of AChE from electric eel (Sigma-Aldrich, St. Louis, MO, USA; 0.22 U/mL in buffer B), 50 µL of buffer B (50 mM Tris–HCl, pH 7.9, containing 0.1% bovine serum albumin), and 25 µL of the test solutions were incubated at 37 °C for 30 min. Subsequently, 125 µL of Ellman’s reagent (3 mM 5,5′-dithiobis(2-nitrobenzoic acid)) prepared in buffer A (50 mM Tris–HCl, pH 7.9, containing 0.10 M NaCl and 0.02 M MgCl_2_ × 6H_2_O) and 25 µL of acetylthiocholine iodide (15 mM) were added to each well to initiate the reaction. The increase in absorbance, corresponding to the formation of 5-thio-2-nitrobenzoate anion, was monitored at 405 nm at 15 s intervals over a 15 min period using a Halo LED96 microplate reader (Dynamica Scientific Ltd., Livingston, UK). Stock solutions of compounds **1**–**5** in MeOH were diluted with buffer B to prepare the test solutions (the ratio between MeOH and buffer B was fixed at 1:9 (*v*/*v*)). Each compound was tested at three different concentrations (50, 20, and 4 µM). The maximum test concentration was limited by compound solubility in the assay buffer (final MeOH content ≤ 1%, *v*/*v*). Wells were visually inspected, and higher concentrations that produced precipitation were excluded from biological evaluation. A negative control was included, consisting of 10% MeOH in buffer B (final MeOH concentration in the well: 1% (*v*/*v*)). As a positive control, rivastigmine (TCI, Tokyo, Japan) was tested at seven different concentrations (ranging from 100 to 1.56 µM). The reaction rate (slope) for each compound at each concentration was determined from the absorbance vs. reaction time data, and the inhibition percentage was calculated using the following equation:

Inhibition [%] = (*v*_0_ − *v_i_*)/*v*_0_ × 100

where *v*_0_ is the initial reaction rate for the negative control and *v_i_* is the reaction rate in the presence of the test compound.

The concentration of rivastigmine was plotted against the percentage inhibition to determine the inhibitor concentration corresponding to 50% enzyme inhibition (IC_50_). IC_50_ value was obtained using a sigmoidal dose–response model with the variable slope fitted to the results. All statistics were performed using GraphPad Prism (GraphPad Software Inc., La Jolla, CA, USA, version 8.0.2). Each experiment was performed in triplicate and repeated three times for validation.

#### 3.6.2. Urease Inhibitory Activity

Urease inhibitory activity was evaluated using a slightly modified version of the previously described Berthelot assay [27,28,29]. Briefly, 70 μL of jack bean urease (Sigma-Aldrich, St. Louis, MO, USA; 2.86 U/mL) in phosphate buffer (50 mM, pH 7.0) was mixed with 10 μL of the test compound solution and pre-incubated at 37 °C for 10 min. Subsequently, 20 μL of 50 mM urea solution (in phosphate buffer, pH 7.0) was added, and the mixture was incubated for an additional 10 min at 37 °C. Thereafter, 45 μL of solution A [an aqueous solution of 1% phenol (*w*/*v*) and 0.005% sodium nitroprusside (*w*/*v*)] and 70 μL of solution B [an aqueous solution of 0.5% NaOH (*w*/*v*) and 0.1% NaOCl (*w*/*v*)] were added. The mixture was shaken for 1 min and incubated for a further 10 min at 37 °C.

Following incubation, the absorbance was measured at 620 nm using a Halo LED96 microplate reader to quantify the formation of ammonium ions. Stock solutions of compounds **1**–**5** (21.5 mM) were prepared in EtOH and further diluted to prepare the test solutions. Each compound was tested at three concentrations: 100, 50, and 25 μM. The maximum test concentrations were limited by compound solubility in the assay buffer (final EtOH content ≤ 4.6%, *v*/*v*); wells were visually inspected, and higher concentrations that produced precipitation were excluded from biological evaluation. EtOH (vehicle) served as the negative control, while phosphate buffer (50 mM, pH 7.0) alone was used as the blank. All assay wells, including controls, were vehicle-matched to the highest EtOH concentration used in the experiment (4.6%, *v*/*v*). Preliminary control experiments confirmed that this concentration of EtOH did not significantly affect urease activity compared with buffer-only controls. A positive control, acetohydroxamic acid (AHA; Sigma-Aldrich, St. Louis, MO, USA), was tested at seven concentrations ranging from 12.5 to 200 μM. The percentage of urease inhibition was calculated using the following equation:

Inhibition [%] = ((A_0_ − A_i_)/A_0_) × 100

where A_0_ is the absorbance of the negative control and A_i_ is the absorbance in the presence of the test compound.

To determine the IC_50_ value of AHA, the percentage inhibition was plotted against the inhibitor concentration, and the data were fitted using a sigmoidal dose–response model with a variable slope. Statistical analyses were performed using GraphPad Prism (GraphPad Software Inc., La Jolla, CA, USA). All experiments were performed in triplicate and repeated three independent times for reproducibility.

### 3.7. Statistical Analysis

Results are presented as mean ± standard deviation (SD). Experiments were performed independently three times, with each experiment conducted in technical triplicate. Technical replicates were averaged before statistical analysis, and the resulting means from the independent experiments were used as individual data points (*n* = 3). Statistical significance was assessed by one-way analysis of variance (ANOVA) followed by Dunnett’s multiple comparisons test (GraphPad Prism, GraphPad Software Inc., La Jolla, CA, USA). Differences were considered statistically significant at *p* < 0.05.

## 4. Conclusions

The phytochemical reinvestigation of *Lycopus europaeus* fruits led to the isolation and characterization of three previously undescribed aromatic abietane diterpenoids, 4-epileonubiastrin (**2**), 3α-acetoxyeuroabienol (**3**), and 11-deoxyeuroabienol (**4**), together with the known fruit metabolite euroabienol (**1**). The structures and relative configurations of these closely related compounds were established by MS, HRMS, IR, and 1D/2D NMR spectroscopy. Manual iterative full-spin analysis proved essential for resolving complex spin systems, enabling the accurate determination of ^1^H chemical shifts and coupling constants and providing strong support for the conformational and configurational assignments within the densely functionalized abietane framework.

Preliminary enzyme screening showed only modest AChE and urease inhibition within the accessible concentration range. Compound **2** was the most active AChE inhibitor in the series, whereas compound **4** was the only metabolite showing a clear dose-dependent effect against jack bean urease. Since inhibition did not reach 50% for any tested compound, the biological results should be viewed as exploratory rather than as evidence of pronounced potency. Overall, the study expands the known diterpenoid profile of *L. europaeus* fruits and highlights these organs as a source of structurally distinctive aromatic abietanes, while also illustrating the value of ^1^H NMR simulation as a complementary tool in natural-product structure elucidation.

## Figures and Tables

**Table 1:** ^1^H NMR spectroscopic data (400 MHz, CDCl_3_) for compounds **1**–**4**.

Position (Integral) ^1^	δ_H_ [ppm] (m, *J* [Hz])			
	1	2	3	4
**1** (1H)	/ ^2^	8.3540 (d, *J* = 10.35)	/ ^2^	/ ^2^
**1β** (1H)	6.3050 (ddd, *J* = 3.70, 2.20, 0.60)	/ ^2^	6.1283 (ddd, *J* = 4.40, 4.10, 0.70)	5.47 (m) ^3^
**2** (1H)		6.0518 (d, *J* = 10.35)		
**2α** (1H)	1.9431 (dddd, *J* = −15.40, 4.50, 3.70, 3.20)	/ ^2^	2.2845 (ddd, *J* = −15.90, 4.90, 4.10)	1.92 (m) ^3^
**2β** (1H)	2.1185 (dddd, *J* = −15.40, 13.70, 4.30, 2.20)	/ ^2^	2.3747 (ddd, *J* = −15.90, 4.40, 3.75)	2.18 (m) ^3^
**3α** (1H)	2.1710 (ddd, *J* = −13.80, 13.70, 4.50)	/ ^2^	/ ^2^	2.17 (m) ^3^
**3β** (1H)	1.4824 (dddd, *J* = −13.80, 4.30, 3.20, 0.60)	/ ^2^	5.1290 (ddd, *J* = 4.90, 3.75, 0.70)	1.55 (m) ^3^
**5α** (1H)	3.2206 (br d, *J* = 1.35)	3.5846 (d, *J* = 1.30)	3.6076 (br d, *J* = 0.90)	3.1831 (br d, *J* = 1.45)
**6α** (1H)	5.0525 (br dd, *J* = 2.20, 1.35)	5.0628 (br dd, *J* = 2.40, 1.30)	5.4965 (br dd, *J* = 2.20, 0.90)	5.1183 (br dd, *J* = 2.10, 1.45)
**7β** (1H)	4.4740 (br dd, *J* = 2.20, −0.55)	4.6033 (br dd, *J* = 2.40, −0.45)	4.5510 (br dd, *J* = 2.20, −0.45)	4.5195 (br dd, *J* = 2.10, −0.50)
**11** (1H)	/ ^2^	/ ^2^	/ ^2^	7.0592 (d, *J* = 8.30)
**12** (1H)	6.5346 (d, *J* = 1.80)	6.6450 (d, *J* = 1.80)	6.5580 (d, *J* = 1.80)	7.1445 (dd, *J* = 8.30, 2.00)
**14** (1H)	6.7855 (dd, *J* = 1.80, −0.55)	6.8405 (dd, *J* = 1.80, −0.45)	6.8069 (dd, *J* = 1.80, −0.45)	7.1770 (dd, *J* = 2.00, −0.50)
**15** (1H)	2.7908 (tt, *J* = 6.95, 6.87)	2.8237 (tt, *J* = 7.00, 6.80)	2.8096 (tt, *J* = 7.00, 6.80)	2.8703 (tt, *J* = 7.00, 6.90)
**16** (3H)	1.2013 (d, *J* = 6.87)	1.2252 (d, *J* = 6.80)	1.2160 (d, *J* = 6.80)	1.2287 (d, *J* = 7.00)
**17** (3H)	1.1993 (d, *J* = 6.95)	1.2250 (d, *J* = 7.00)	1.2132 (d, *J* = 7.00)	1.2284 (d, *J* = 6.90)
**18α**	/ ^2^	/ ^2^	/ ^2^	/ ^2^
**19β** (3H)	1.4587 (s)	1.6324 (s)	1.4920 (s)	1.4557 (s)
**20β** (3H)	1.7404 (s)	1.8248 (s)	1.7615 (s)	1.5760 (s)
**1α-OAc** (3H)	1.8090 (s)	/ ^2^	1.7599 (s)	1.7598 (s)
**3α-OAc** (3H)	/ ^2^	/ ^2^	1.9446 (s)	/ ^2^
**6β-OAc** (3H)	2.0322 (s)	2.0442 (s)	2.0180 (s)	2.0320 (s)
**18α-COOMe** (3H)	3.7605 (s)	3.7038 (s)	3.6983 (s)	3.7590 (s)
**7α-OH** (1H)	2.6800 (br s)	2.7100 (br s)	2.1800 (br s)	2.6600 (br s)
**11-OH** (1H)	6.0170 (s)	6.1780 (s)	6.2090 (s)	/ ^2^

^1^ The carbon atom numbering scheme for the abietane skeleton is depicted in structure **1** of Figure 1. ^2^ No proton is present at this position. ^3^ The chemical shifts in the protons were estimated based on correlations observed in the HSQC spectrum.

**Table 2:** ^13^C NMR spectroscopic data (100.6 MHz, CDCl_3_) for compounds **1**–**4**.

Position ^1^	δ_C_ [ppm]			
	1	2	3	4
**1**	74.8	159.7	73.9	74.7
**2**	22.3	125.7	27.2	22.6
**3**	31.5	197.9	73.9	31.8
**4**	47.8	58.4	50.3	47.2
**5**	37.4	42.0	34.2	37.0
**6**	75.3	73.6	74.1	75.8
**7**	70.4	69.8	70.4	70.1
**8**	136.3	135.9	136.3	133.5
**9**	127.7	125.4	126.8	141.4
**10**	42.7	40.3	42.4	41.6
**11**	153.5	153.2	153.4	124.2
**12**	114.8	115.2	114.5	127.0
**13**	148.4	149.7	148.7	147.0
**14**	121.9	122.6	122.1	129.0
**15**	33.3	33.5	33.3	33.5
**16**	23.6	23.93 or 23.73 ^2^	23.6	24.1 or 23.8 ^2^
**17**	23.9	23.93 or 23.73 ^2^	24.0	24.1 or 23.8 ^2^
**18**	178.2	173.1	174.6	178.0
**19**	18.4	18.1	18.9	18.3
**20**	21.9	25.7	21.7	27.3
**1α-OAc**	171.6 21.4	/ ^3^	171.3 21.3	170.8 21.2
**3α-OAc**	/	/	169.7 21.1	/
**6β-OAc**	170.6 21.6	170.5 21.5	170.5 22.0	170.6 21.6
**18-COOMe**	52.5	53.1	52.4	52.6

^1^ The carbon atom numbering scheme for the abietane skeleton is depicted in structure **1** of Figure 1. ^2^ It was not possible to unambiguously assign the exact positions of the two methyl carbons in the isopropyl group in the ^13^C NMR spectrum, as their chemical shifts are too similar to be resolved individually. ^3^ No carbon atom is present at this position.

## Data Availability

The original contributions presented in this study are included in the article/Supplementary Materials. Further inquiries can be directed to the corresponding authors.

## Supplementary Materials

The following supporting information can be downloaded at: https://www.mdpi.com/article/10.3390/molecules31142441/s1, Figure S1: MS spectrum of euroabienol (**1**); Figure S2: ^1^H NMR (400 MHz, CDCl_3_) spectrum of euroabienol (**1**); Figure S3: ^13^C NMR (100.6 MHz, CDCl_3_) spectrum of euroabienol (**1**); Figure S4: MS spectrum of 4-epileonubiastrin (**2**); Figure S5: IR spectrum of 4-epileonubiastrin (**2**); Figure S6: ^1^H NMR (400 MHz, CDCl_3_) spectrum of 4-epileonubiastrin (**2**); Figure S7: ^13^C NMR (100.6 MHz, CDCl_3_) spectrum of 4-epileonubiastrin (**2**); Figure S8: NOESY spectrum of 4-epileonubiastrin (**2**) with expansions for the correlations of Me-19 and Me-20; Figure S9: MS spectrum of 3α-acetoxyeuroabienol (**3**); Figure S10: IR spectrum of 3α-acetoxyeuroabienol (**3**); Figure S11: ^1^H NMR (400 MHz, CDCl_3_) spectrum of 3α-acetoxyeuroabienol (**3**); Figure S12: ^13^C NMR (100.6 MHz, CDCl_3_) spectrum of 3α-acetoxyeuroabienol (**3**); Figure S13: MS spectrum of 11-deoxyeuroabienol (**4**); Figure S14: IR spectrum of 11-deoxyeuroabienol (**4**); Figure S15: ^1^H NMR (400 MHz, CDCl_3_) spectrum of 11-deoxyeuroabienol (**4**); Figure S16: ^13^C NMR (100.6 MHz, CDCl_3_) spectrum of 11-deoxyeuroabienol (**4**); Figure S17: MS spectrum of *O*-methylated euroabienol (**5**); Figure S18: IR spectrum of *O*-methylated euroabienol (**5**); Figure S19: ^1^H NMR (400 MHz, CDCl_3_) spectrum of *O*-methylated euroabienol (**5**); Figure S20: ^13^C NMR (100.6 MHz, CDCl_3_) spectrum of *O*-methylated euroabienol (**5**).
