# Supplementary material for: New Aromatic Abietane Diterpenoids from Lycopus europaeus L. Fruits: 1H NMR Simulation-Aided Structure Elucidation and Enzyme Inhibition Screening
Source: Molecules. 2026 Jul 12;31(14):2441. doi: 10.3390/molecules31142441 (PMC13414173; doi:10.3390/molecules31142441)
Supplement: Supplementary file 1 [file molecules-31-02441-s001.zip › molecules-4426161-supplementary.pdf]

Supplementary Material

for

New aromatic abietane diterpenoids from *Lycopus europaeus* L. fruits: ^{1}H NMR simulation-aided structure elucidation and enzyme inhibition screening

Marija S. Genčić*, Danijela N. Nikolić, Jelena D. Živanović, Jelena M. Denić, Niko S. Radulović*

Department of Chemistry, Faculty of Sciences and Mathematics, University of Niš, Višegradska 33, 18000 Niš, Serbia

Table of content:

1. **Figure S1.** MS spectrum of euroabienol (**1**) S2
2. **Figure S2.** ^{1}H NMR (400 MHz, $CDCl_3$) spectrum of euroabienol (**1**) S2
3. **Figure S3.** ^{13}C NMR (100.6 MHz, $CDCl_3$) spectrum of euroabienol (**1**) S3
4. **Figure S4.** MS spectrum of 4-epileonubiastrin (**2**) S4
5. **Figure S5.** IR spectrum of 4-epileonubiastrin (**2**) S4
6. **Figure S6.** ^{1}H NMR (400 MHz, $CDCl_3$) spectrum of 4-epileonubiastrin (**2**) S5
7. **Figure S7.** ^{13}C NMR (100.6 MHz, $CDCl_3$) spectrum of 4-epileonubiastrin (**2**) S5
8. **Figure S8.** NOESY spectrum of 4-epileonubiastrin (**2**) with expansions for the correlations of Me-19 and Me-20 S6
9. **Figure S9.** MS spectrum of 3α-acetoxyeuroabienol (**3**) S7
10. **Figure S10.** IR spectrum of 3α-acetoxyeuroabienol (**3**) S7
11. **Figure S11.** ^{1}H NMR (400 MHz, $CDCl_3$) spectrum of 3α-acetoxyeuroabienol (**3**) S8
12. **Figure S12.** ^{13}C NMR (100.6 MHz, $CDCl_3$) spectrum of 3α-acetoxyeuroabienol (**3**) S8
13. **Figure S13.** MS spectrum of 11-deoxyeuroabienol (**4**) S9
14. **Figure S14.** IR spectrum of 11-deoxyeuroabienol (**4**) S9
15. **Figure S15.** ^{1}H NMR (400 MHz, $CDCl_3$) spectrum of 11-deoxyeuroabienol (**4**) S10
16. **Figure S16.** ^{13}C NMR (100.6 MHz, $CDCl_3$) spectrum of 11-deoxyeuroabienol (**4**) S10
17. **Figure S17.** MS spectrum of *O*-methylated euroabienol (**5**) S11
18. **Figure S18.** IR spectrum of *O*-methylated euroabienol (**5**) S11
19. **Figure S19.** ^{1}H NMR (400 MHz, $CDCl_3$) spectrum of *O*-methylated euroabienol (**5**) S12
20 **Figure S20.** ^{13}C NMR (100.6 MHz, $CDCl_3$) spectrum of *O*-methylated euroabienol (**5**) S12

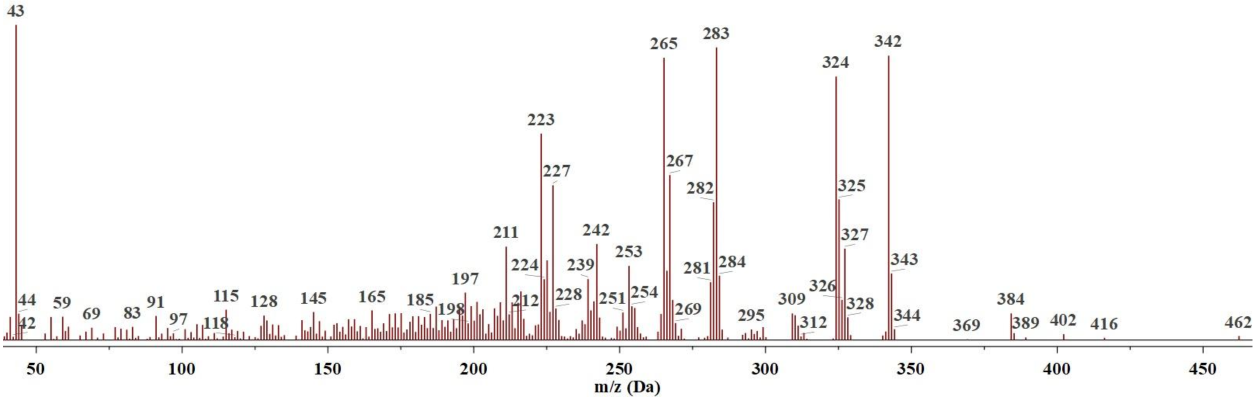

Figure S1. MS spectrum of euroabienol (**1**)

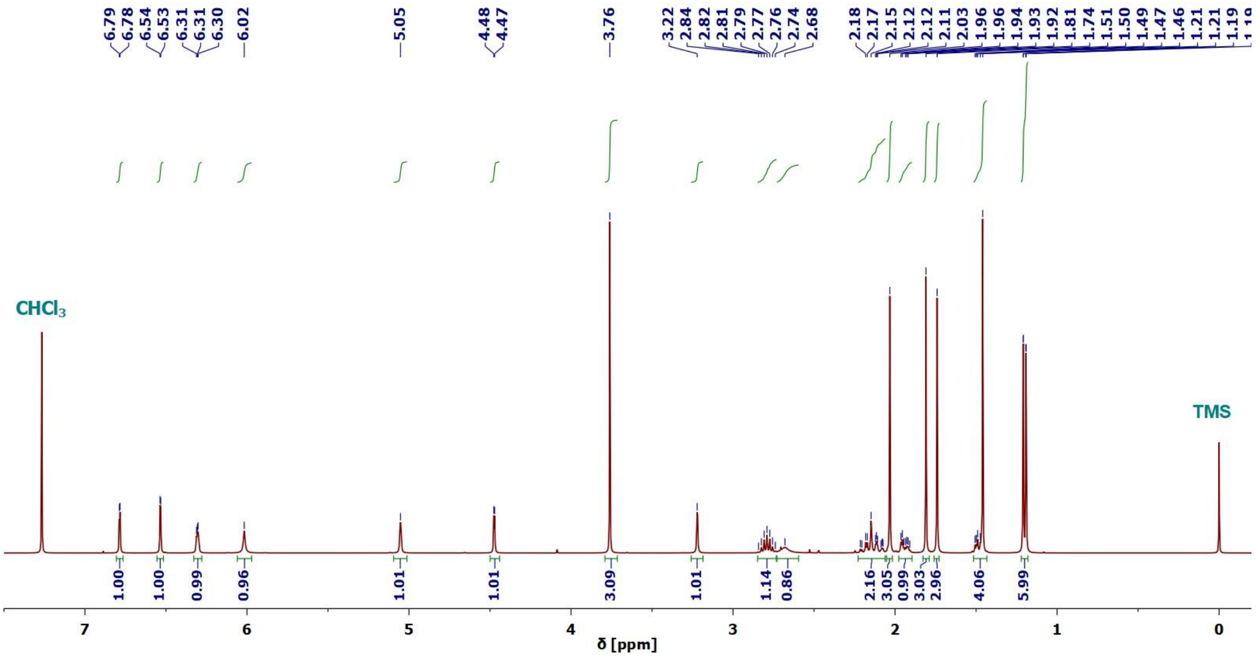

Figure S2. ^{1}H NMR (400 MHz, $CDCl_3$) spectrum of euroabienol (**1**)*

* All spectra acquired in $CDCl_3$ at 20 °C; referenced to TMS.

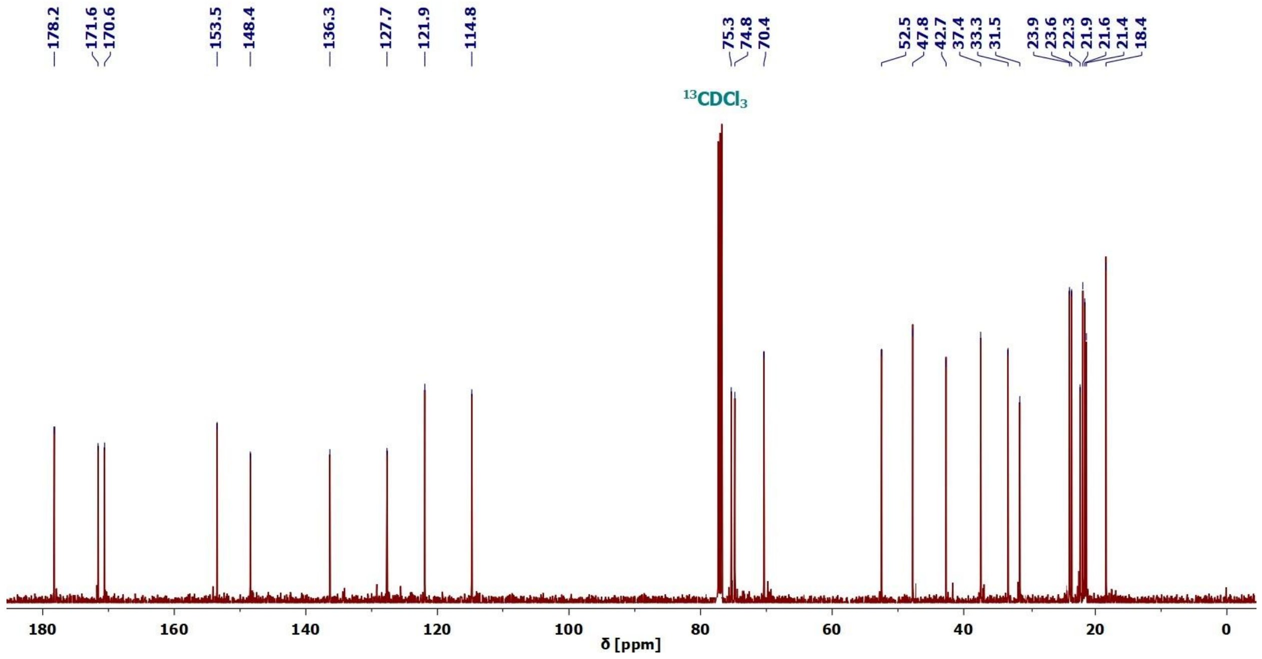

Figure S3. ^{13}C NMR (100.6 MHz, $CDCl_3$) spectrum of euroabienol (**1**)

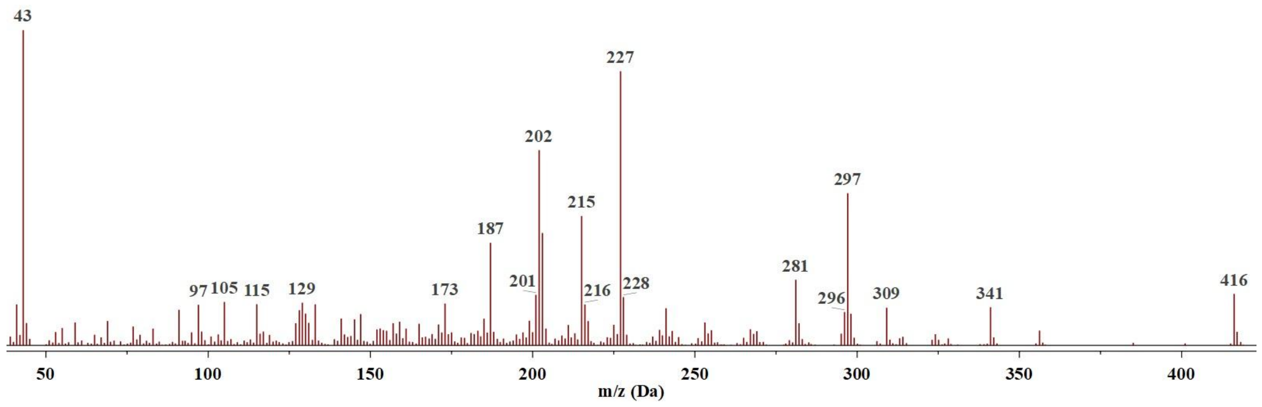

Figure S4. MS spectrum of 4-epileonubiastrin (**2**)

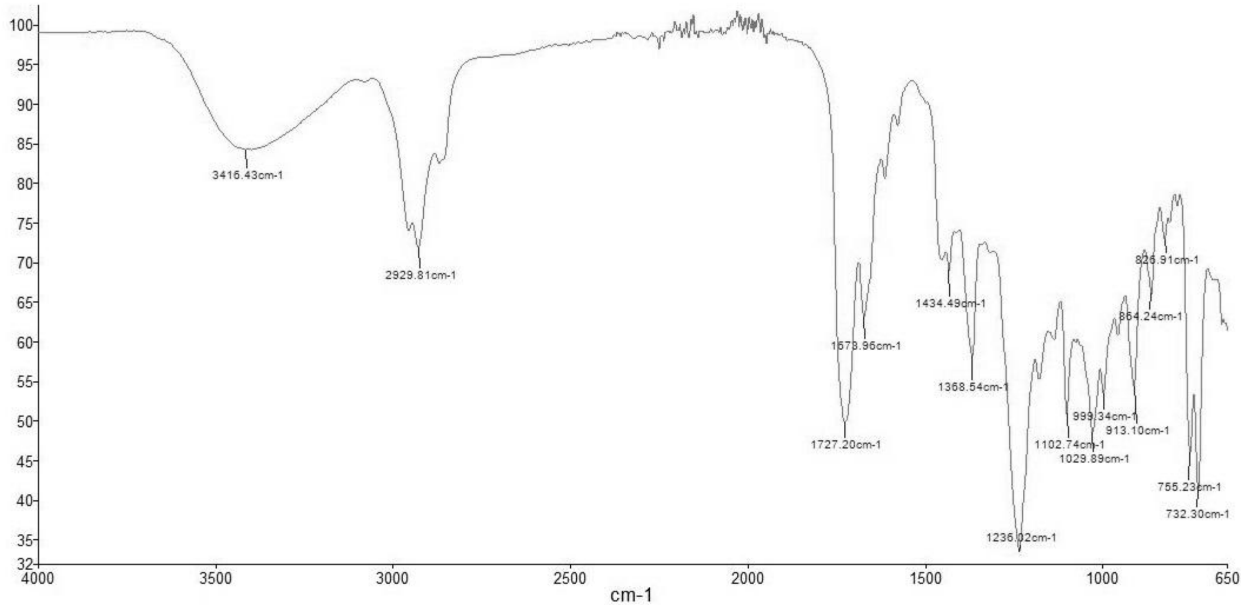

Figure S5. IR spectrum of 4-epileonubiastrin (**2**)

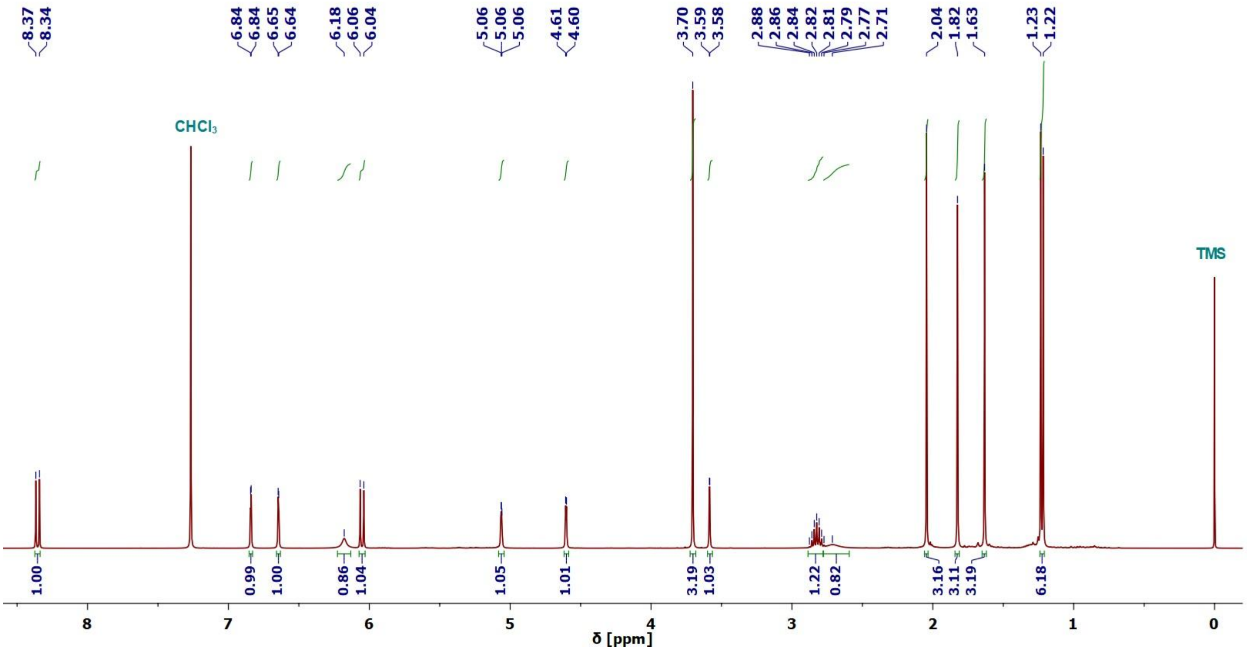

Figure S6. ^{1}H NMR (400 MHz, $CDCl_3$) spectrum of 4-epileonubiastrin (**2**)

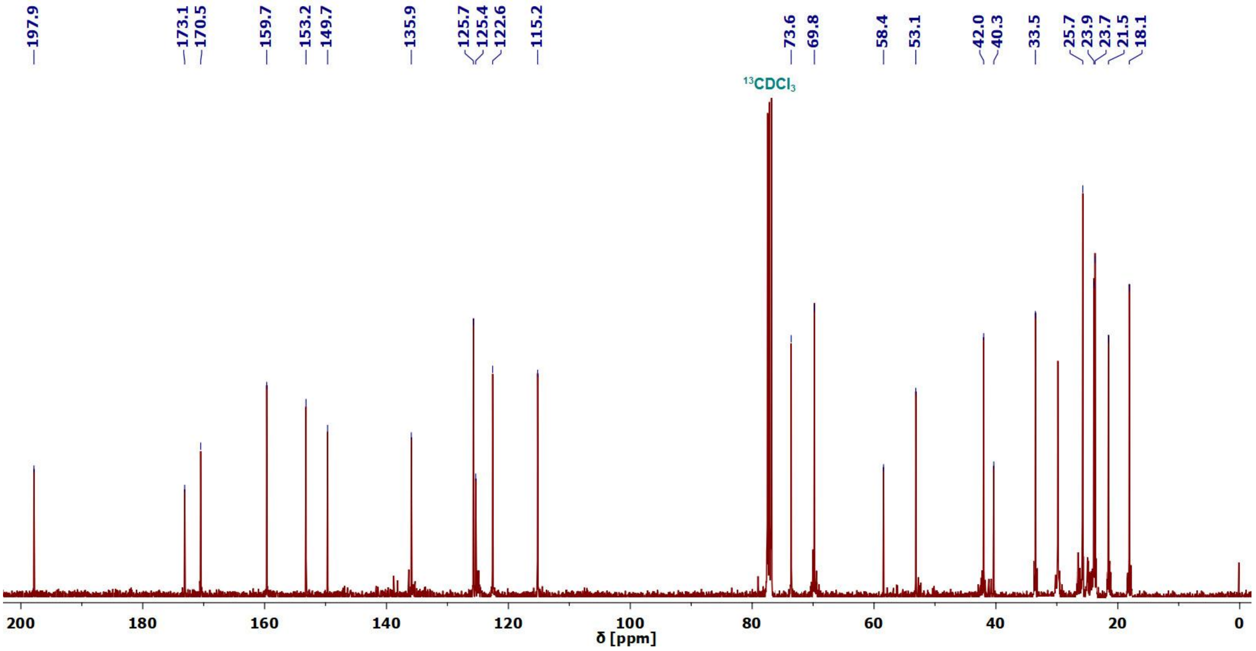

Figure S7. ^{13}C NMR (100.6 MHz, $CDCl_3$) spectrum of 4-epileonubiastrin (**2**)

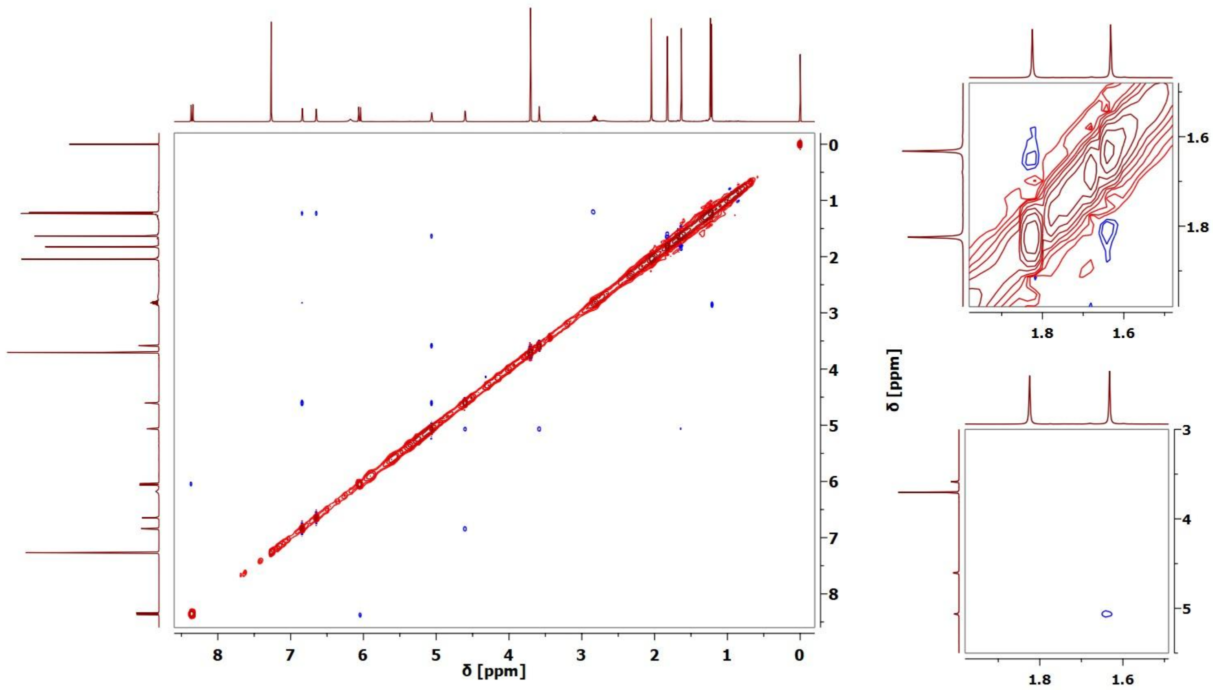

Figure S8. NOESY spectrum of 4-epileonubiastrin (**2**) with expansions for the correlations of Me-19 and Me-20

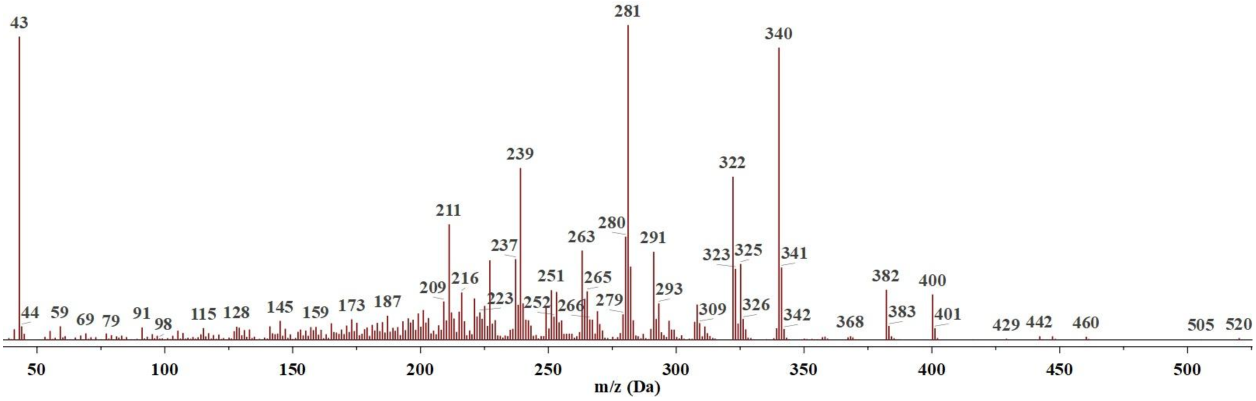

Figure S9. MS spectrum of 3α-acetoxyeuroabienol (**3**)

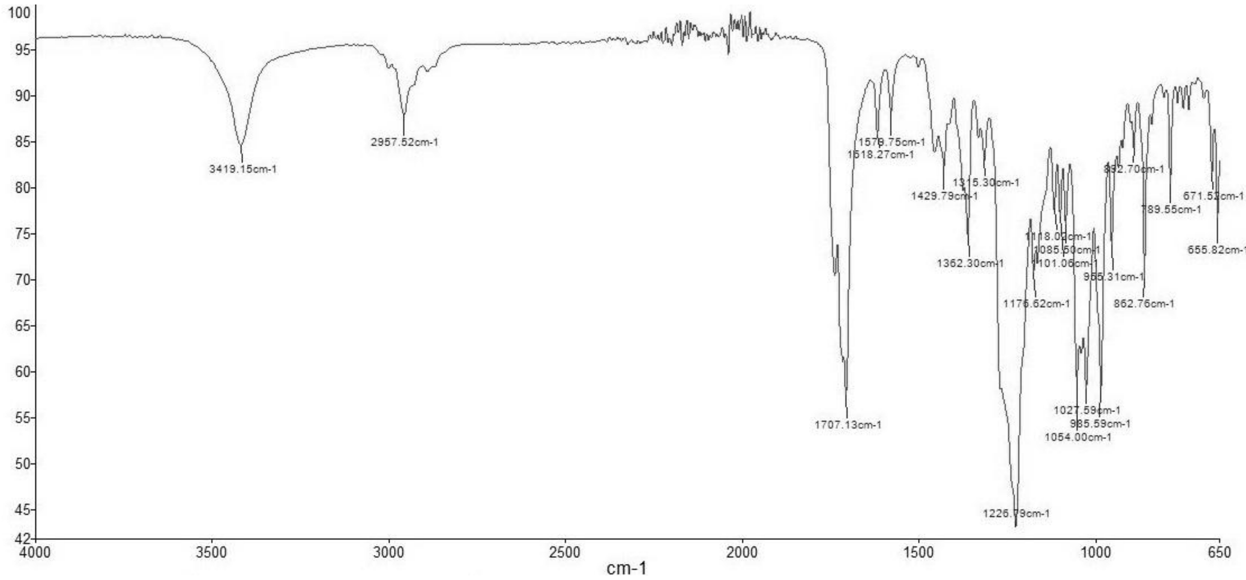

Figure S10. IR spectrum of 3α-acetoxyeuroabienol (**3**)

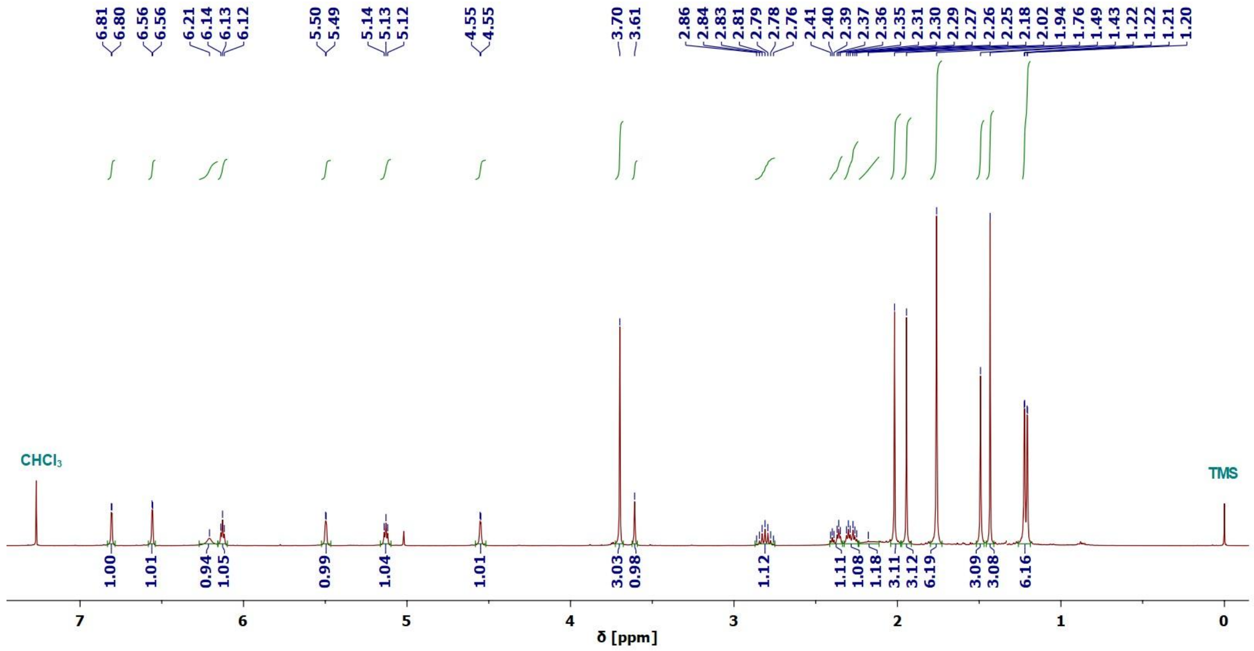

Figure S11. ^{1}H NMR (400 MHz, $CDCl_3$) spectrum of 3α-acetoxyeuroabienol (**3**)

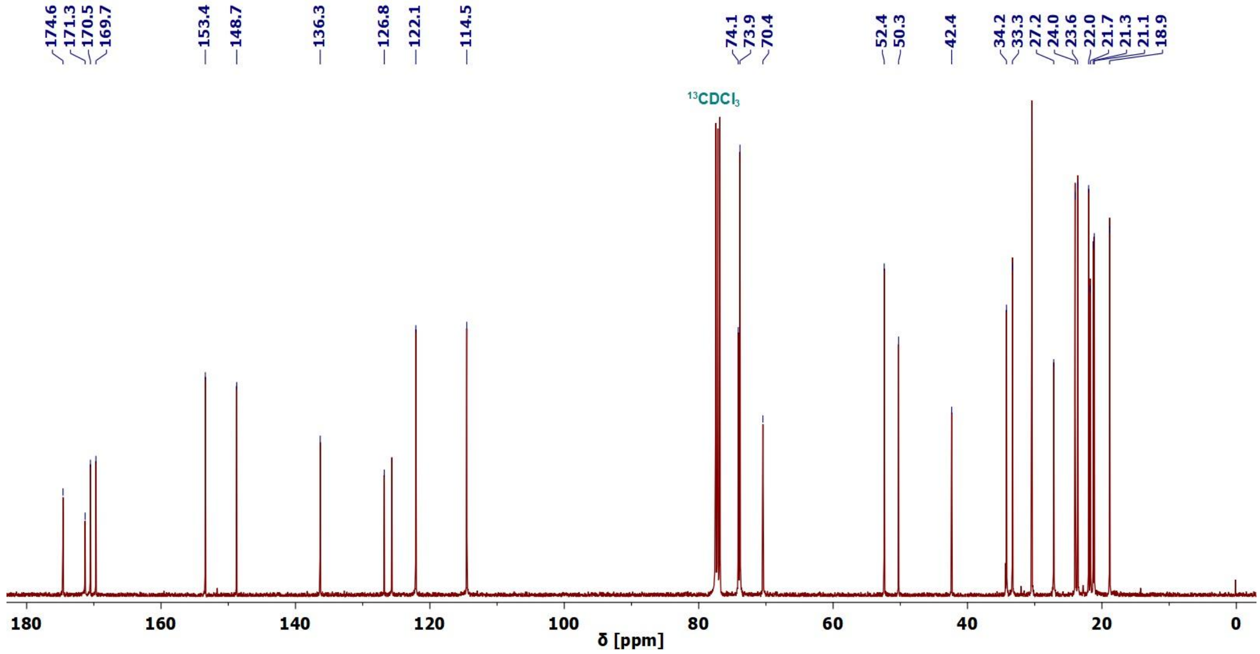

Figure S12. ^{13}C NMR (100.6 MHz, $CDCl_3$) spectrum of βα-acetoxyeuroabienol (**3**)

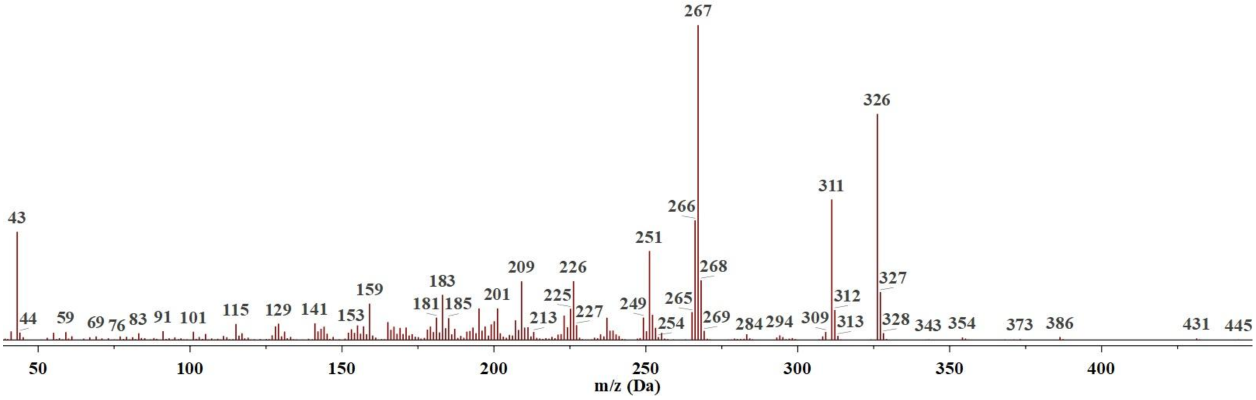

Figure S13. MS spectrum of 11-deoxyeuroabienol (**4**)

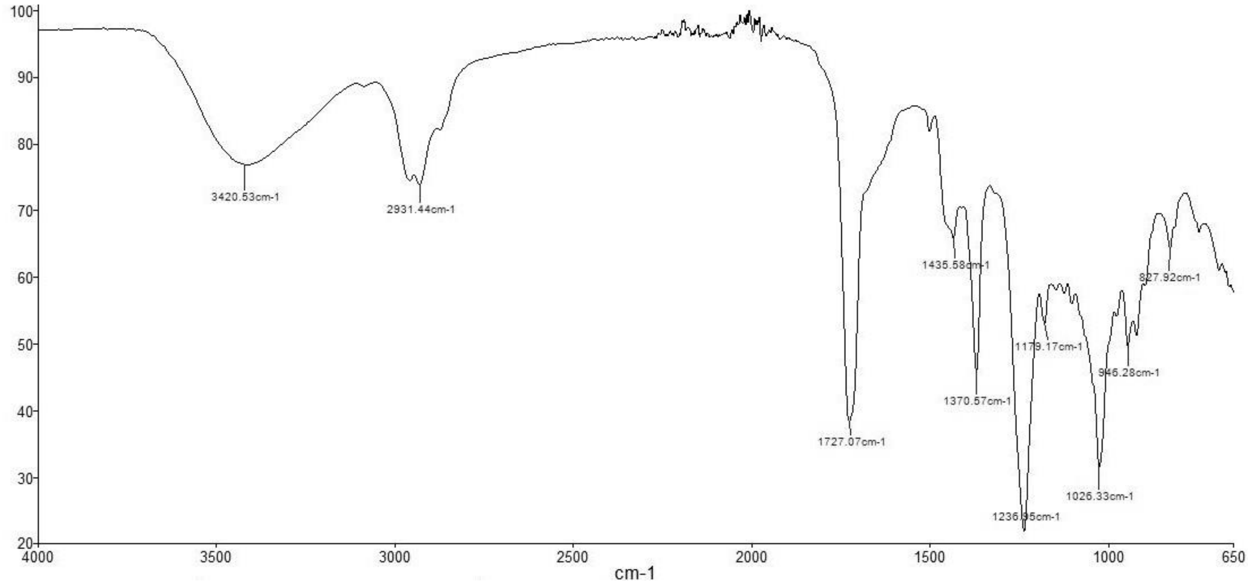

Figure S14. IR spectrum of 11-deoxyeuroabienol (**4**)

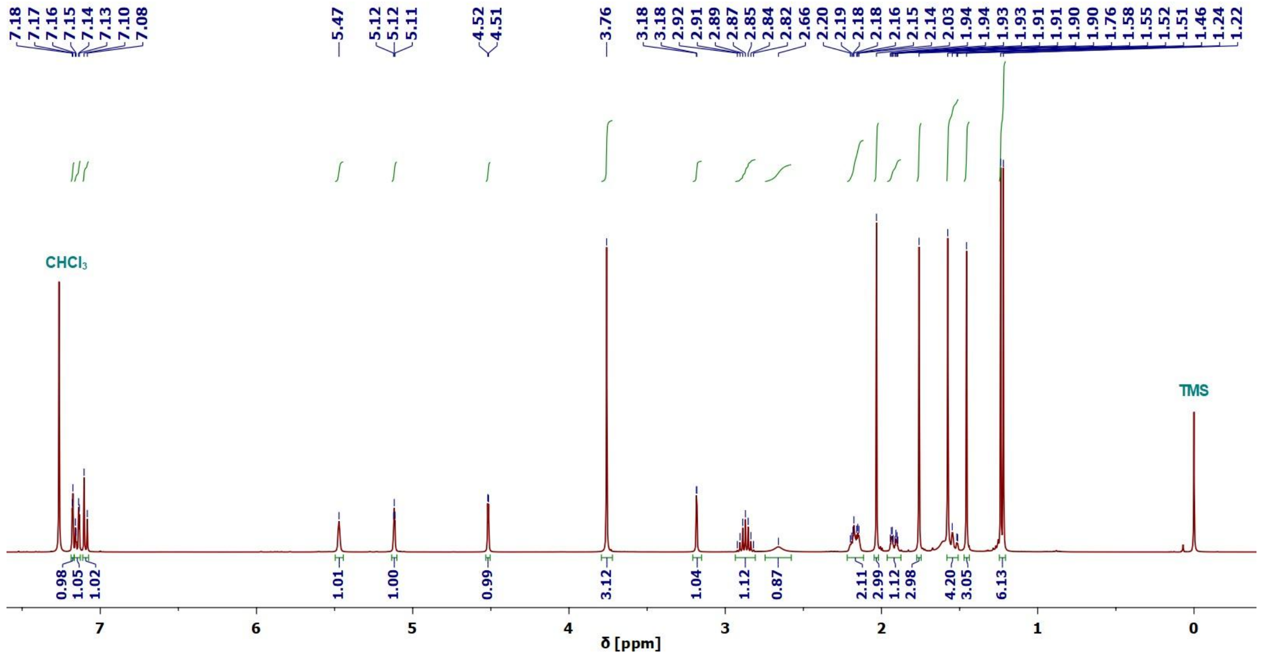

Figure S15. ^{1}H NMR (400 MHz, $CDCl_3$) spectrum of 11-deoxyeuroabienol (**4**)

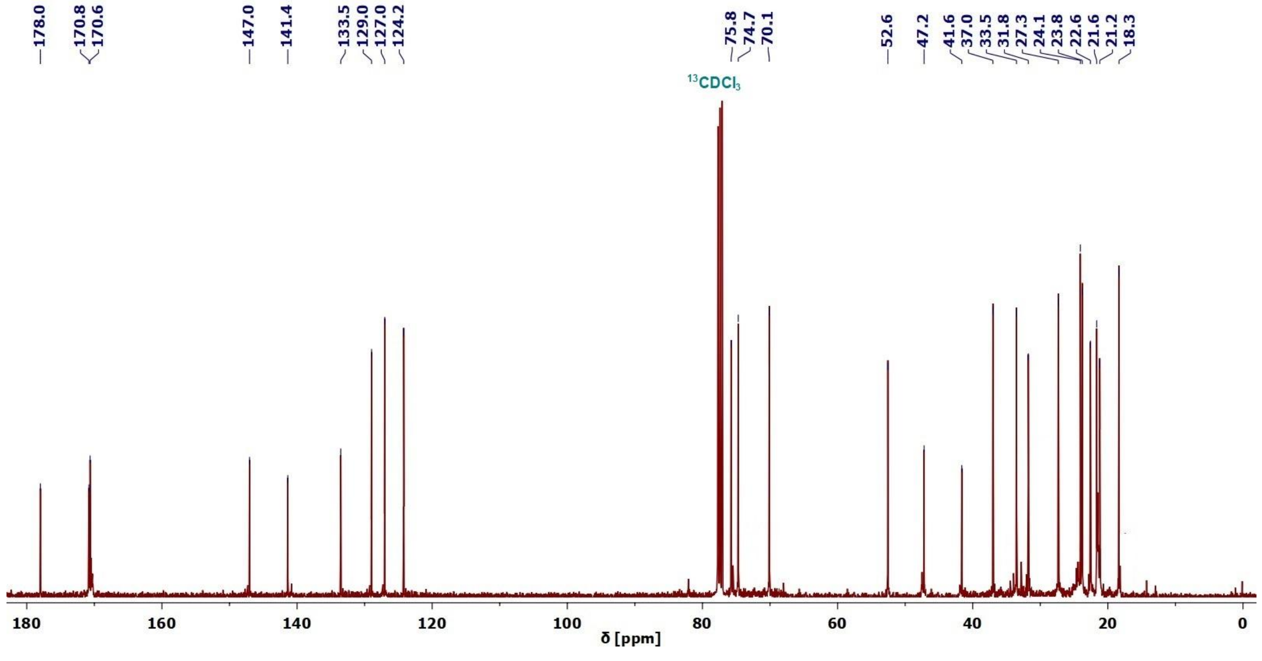

Figure S16. ^{13}C NMR (100.6 MHz, $CDCl_3$) spectrum of 11-deoxyeuroabienol (**4**)

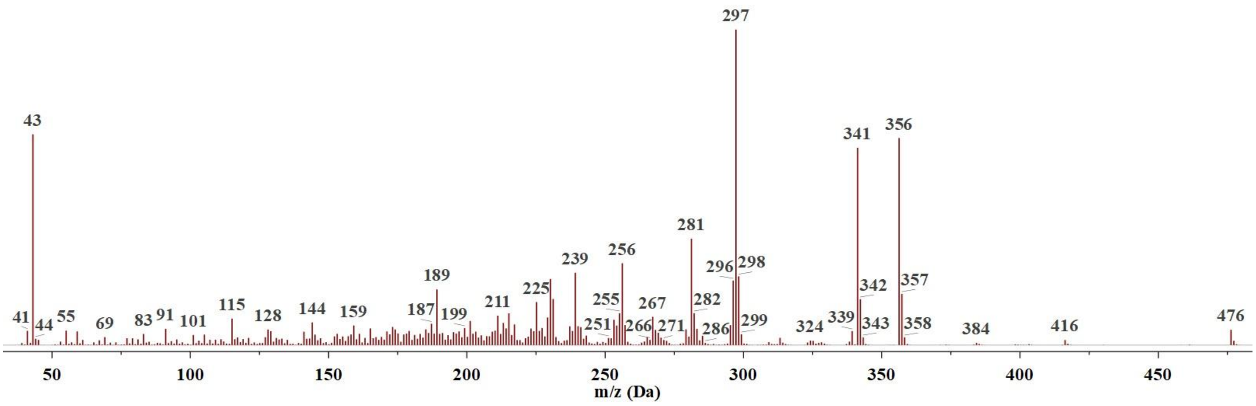

Figure S17. MS spectrum of *O*-methylated euroabienol (**5**)

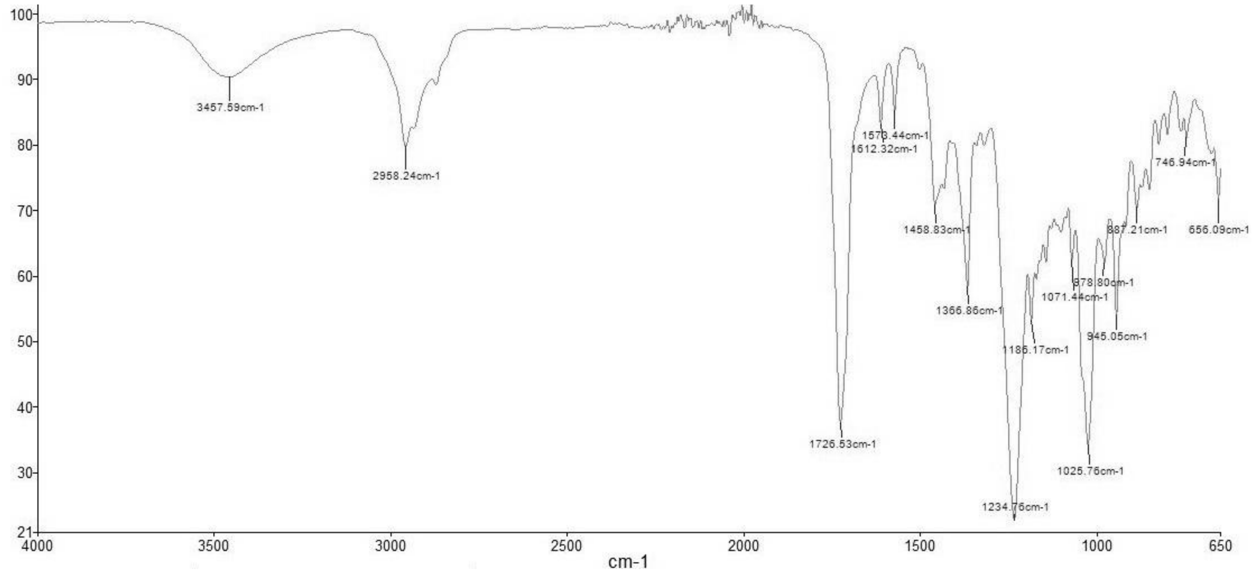

Figure S18. IR spectrum of *O*-methylated euroabienol (**5**)

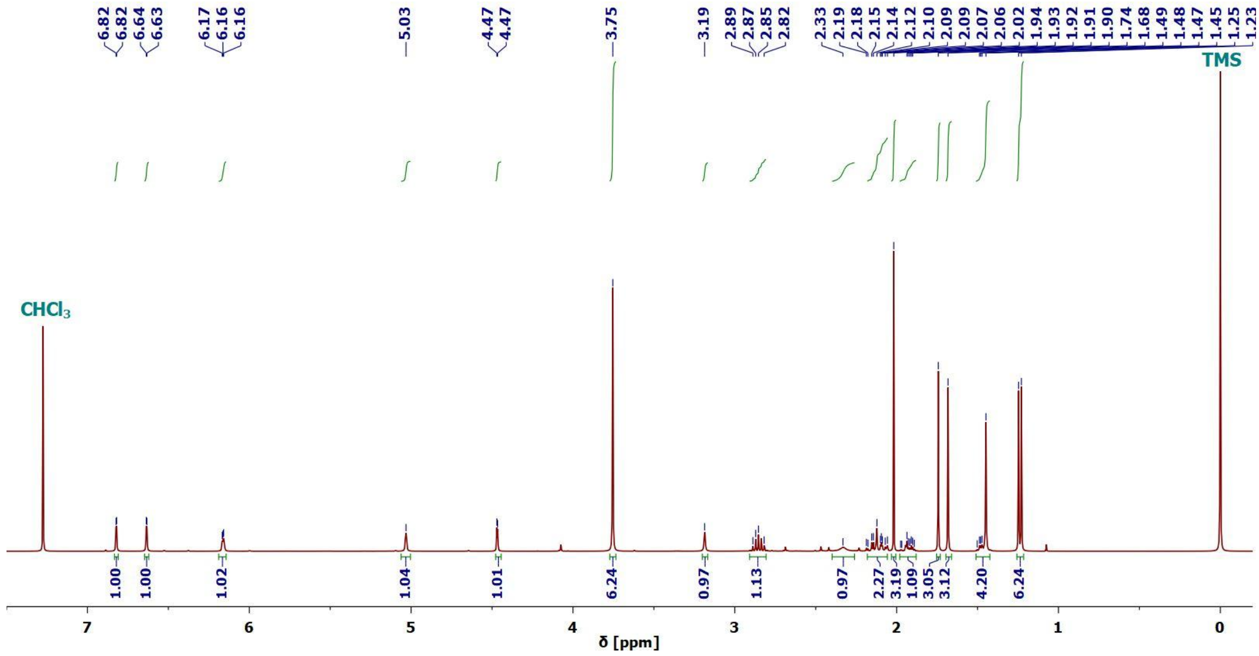

Figure S19. ^{1}H NMR (400 MHz, $CDCl_3$) spectrum of *O*-methylated euroabienol (**5**)

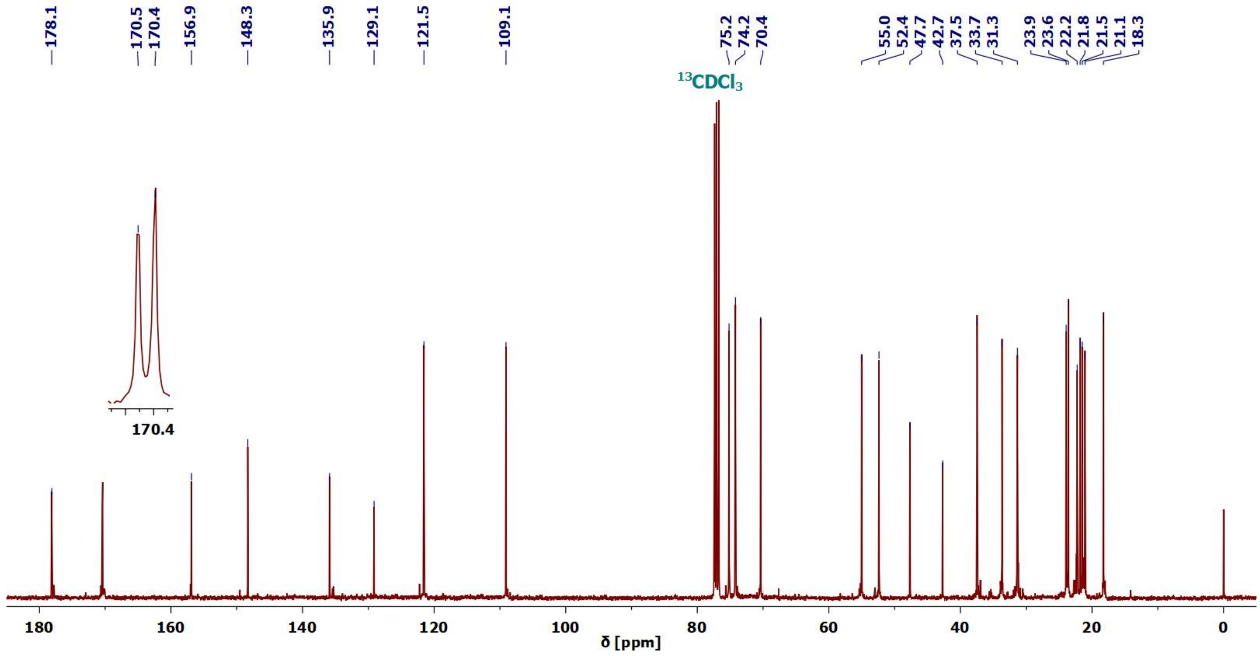

Figure S20. ^{13}C NMR (100.6 MHz, $CDCl_3$) spectrum of *O*-methylated euroabienol (**5**)
